# Impact of a guideline-based best practice alert on pneumococcal vaccination rates in adults in a primary care setting

**DOI:** 10.1186/s12913-019-4263-2

**Published:** 2019-07-10

**Authors:** Carrie McAdam-Marx, Casey Tak, Tanaz Petigara, Nathan W. Jones, Minkyoung Yoo, Melissa Struwe Briley, Karen Gunning, Lisa Gren

**Affiliations:** 1 Department of Pharmacotherapy, University of Utah, Salt Lake City, UT USA; 2 Division of Pharmaceutical Evaluation & Policy, University of Arkansas for Medical Sciences, 4301 W. Markham St, Little Rock, AR 72205 USA; 3 Division of Pharmaceutical Outcomes and Policy, University of North Carolina at Chapel Hill, Chapel Hill, NC USA; 4 Merck & Co., Inc, Kenilworth, NJ USA; 5 Division of Public Health, University of Utah, Salt Lake City, UT USA; 6 Department of Economics, University of Utah, Salt Lake City, UT USA; 7 University of Utah Health, Salt Lake City, UT USA; 8 Department of Family & Preventive Medicine, University of Utah, Salt Lake City, UT USA

**Keywords:** Pneumonia, pneumococcal, Pneumococcal infections, Risk factors, Immunocompromised patients, Health resources, Health care costs

## Abstract

**Background:**

Despite the high burden of pneumococcal disease, pneumococcal vaccine coverage continues to fall short of Healthy People 2020 goals. A quasi-experimental design was used to investigate the impact of pneumococcal-specific best-practice alerts (BPAs) with and without workflow redesign compared to health maintenance notifications only, on pneumococcal vaccination rates in at-risk and high-risk adults, and on series completion in immunocompetent adults aged 65+ years.

**Methods:**

This retrospective study used electronic health record and administrative data to identify pneumococcal vaccinations using cross sectional and historical cohorts of adults age 19+ years from 2013 to 2017 who attended clinics associated with the University of Utah Health. Difference-in-differences (DD) analyses was used to assess the impact of interventions across three observation periods (Baseline, Interim, and Follow Up). Adherence to the 2-dose vaccination schedule in older adults was measured through a longitudinal analysis.

**Results:**

In DD analyses, implementing both workflow redesign and the BPA raised the vaccination rate by 8 percentage points (pp) (*P* < 0.001) and implementing the BPA only raised the rate by 7 pp. (P < 0.001) among at-risk adults age 19–64 years, relative to implementing health maintenance notifications (i.e., usual care) only in comparison clinics. In high-risk adults age 19–64 years, the BPA with or without workflow redesign did not significantly affect vaccination rates from baseline to follow up relative to health maintenance notifications. Per DD analyses, the effect of the BPA was mixed in immunocompetent and immunocompromised adults age 65+ years. However, immunocompetent older adults attending a clinic that implemented the BPA plus health maintenance notifications and workflow redesign (all 3 interventions) had 1.94 times higher odds (Odds ratio (OR) 1.94; *P* = 0.0003, 95% CI 1.24, 3.01) to receive the second pneumococcal dose than patients attending a usual practice clinic (i.e., no intervention).

**Conclusions:**

A pneumococcal BPA tool that reflects current guidelines implemented with and without workflow redesign improved vaccination rates for at-risk adults age 19–64 years and increased the likelihood of adults aged 65+ to complete the recommended 2-dose series. However, in other adult patient groups, the BPA was not consistently associated with improvements in pneumococcal vaccination rates.

**Electronic supplementary material:**

The online version of this article (10.1186/s12913-019-4263-2) contains supplementary material, which is available to authorized users.

## Background

Pneumococcal disease is a leading cause of morbidity and mortality, especially among immunocompromised individuals and older adults. [1] Annual total direct and indirect costs associated with pneumococcal disease in the US are estimated to be $5.1 billion. [1] Guidelines for the use of approved pneumococcal vaccines in the US have been developed by the Advisory Committee on Immunization Practices (ACIP). [2, 3]

Since 1997, the ACIP has recommended a single dose of the 23-valent pneumococcal polysaccharide vaccine (PPSV23) for adults aged 19–64 years with certain chronic conditions (such as chronic heart disease, chronic lung disease and diabetes) that put them at risk for pneumococcal infections and/or poor outcomes from an infection (hereafter referred to as at-risk adults). [2] In 2012, the 13-valent pneumococcal conjugate vaccine (PCV13) followed by PPSV23 vaccination 8 weeks later was recommended for vaccine-naïve adults aged 19+ years who may be immunocompromised due to a condition or its treatment such as chronic renal failure, HIV and cancer (hereafter referred to as high-risk adults). [2] Both PCV13 and PPSV23 were recommended for adults aged 65+ in 2014 with updates to recommendations regarding timing of the second dose in 2015 [3] (Additional file 1: Table S1).

Despite the high burden of pneumococcal disease, pneumococcal vaccine coverage continues to fall short of Healthy People 2020 goals (60% among high-risk adults aged 19–64 years and 90% among persons aged 65+). [4] In 2015 in the US, only 23% of at-risk and high-risk adults aged 19–64 years and 64% of adults aged 65+ reported ever receiving a pneumococcal vaccination in their lifetime. [5]

Clinical decision support tools can support health care providers to identify patients who are eligible for recommended preventive services. [6] One such tool is an electronic medical record-based clinical alert system, or Best Practice Alert (BPA). A BPA triggers a patient-specific alert when a set of criteria is met, providing guidance regarding specific health needs that can be considered during the visit. For vaccinations, BPAs can assist in identifying appropriate patients, update a patient’s vaccination history in the electronic medical record if unavailable, and document reasons for vaccination refusal. [6–8]

Electronic medical record-based alerts or reminders targeted towards healthcare providers have been shown to be effective in improving vaccine coverage and documentation rates in children, adolescents and adults. Following BPA implementation in one academic institution and one community practice clinic, the influenza and pneumococcal vaccination rates increased from 47 to 65% and 19 to 41% (*P* < 0.001 for both) respectively. [7] Similarly, an evaluation of an electronic medical record-based vaccination reminder at 4 community pediatric clinics found that the number of eligible children who remained unvaccinated was significantly lower at all clinics when the reminder was switched on compared to when the reminder was switched off (adjusted RR 0.90 [0.83–0.98]). [8]

In 2016, the University of Utah Health (U of U Health) developed clinical decision support tools to support the implementation of ACIP pneumococcal vaccination guidelines in all age and risk groups.

Two tools were developed – a passive health maintenance notification and a proactive BPA or pop-up message. The health maintenance screen, which is part of the electronic medical record and is implemented across all clinics, alerts staff to preventative care needs, including vaccinations. Typically, nursing staff pull up and review the health maintenance screen upon rooming the patient. For pneumococcal vaccinations, the notification prompts the staff to discuss the vaccination with patients and includes links to vaccination guidelines and order templates. The pneumococcal BPA sends a pop-up message to the healthcare provider at the time of order entry (for any reason) stating the vaccination need for that patient. The BPA serves as a follow-through reminder to the health maintenance notification, and the decision to implement the tool is made at the clinic level.

Prior to the release of the pneumococcal clinical decision support tools (in 2015), several clinics that also implemented the BPA evaluated and modified their pneumococcal vaccination workflows to fully integrate the BPA as part of their quality improvement efforts. The workflow redesign involved mapping current activities, listing all activities and who performs them, identifying opportunities to improve or streamline processes, and incorporation of the desired improvements, to ultimately achieve the desired process and/or patient outcomes.

The purpose of this study was to assess the impact of health maintenance notifications and pneumococcal-specific BPAs with and without workflow redesign, on pneumococcal vaccination rates in at-risk and high-risk adults (age 19–64 years and age 65+ years) attending U of U Health community-based clinics, and on series completion in immunocompetent adults aged 65+ years.

## Methods

### Study design and data sources

This retrospective study used electronic health record and administrative data to identify pneumococcal vaccinations using cross sectional and historical cohorts from 2013 to 2017 who attended Family Medicine (FM) and Internal Medicine (IM) clinics associated with the U of U Health.

The primary data source for assessing vaccination rates was the U of U Health Enterprise Data Warehouse (EDW). The EDW contains administrative and electronic medical record data for 1.4 million patients treated at the U of U Health from 1990 to present day, including vaccination history from the Utah State Immunization Information System (USIIS). Diagnosis codes and medication orders in the EDW were used to identify adults recommended for pneumococcal vaccination based on age and presence of at-risk or high-risk conditions. Vaccine administration and vaccination history were identified through electronic medical record-based care delivery and billing codes (CPT) for vaccines administered by a U of U Health provider. These data were integrated with the EDW by the implementation of a system-wide, real-time interface in April 2016.

Prior to the launch of the revised clinical decision support tools, a subgroup of four FM clinics undertook a process mapping and redesign exercise as a Quality Improvement initiative. These clinics reviewed their pneumococcal vaccination workflow, identified the most appropriate point of clinical decision support pneumococcal report generation, then proposed and implemented redesigned workflows to integrate the existing clinical decision support pneumococcal reports into routine practice during the first half of the 2015 calendar year. The redesigns were clinic specific, but in general they identified processes to identify patients in need of vaccination prior to the visit, confirm a patient’s vaccination history during the visit, and document vaccine administration details including patient refusal or receipt of vaccination elsewhere. For immunocompromised individuals and older adults (aged 65+) who required sequential PCV13-PPV23 vaccination, an additional step of scheduling the next appointment was incorporated into the workflow.

A pre-test post-test design with a non-equivalent comparison group was used to evaluate the impact of health maintenance notifications, BPAs and clinical workflow redesign on vaccination rates. Three clinic groups were included in the analysis. Four FM Clinics that received the BPA tool, health maintenance notifications, and also implemented workflow redesign to integrate the tools at the appropriate point of care (FM Group A); 7 FM Clinics that received the BPA tool and health maintenance notifications, but did not undertake workflow redesign (FM Group B); and 10 IM Clinics that received health maintenance notifications but did not receive the BPA tool and did not implement any workflow redesign (IM Group C). The IM clinics served as the non-equivalent comparison group.

### Study population

The study population consisted of adults (aged 19+ years) receiving care at a U of U Health FM or IM clinic who met at least 1 of the ACIP criteria for pneumococcal vaccination. At-risk or high-risk adults were aged 19–64 years, while immunocompetent and immunocompromised adults were aged 65 + .

### Measures

Pneumococcal vaccination rates were determined for 3 observation periods (Baseline, Interim, and Follow-up) at all clinics included in the study (Table 1) based on vaccinations given in clinic and documentation of doses administered at other sites of care per patient recall or USIIS. Vaccination rates were calculated in patients who were eligible for pneumococcal vaccination (at-risk/high-risk adults or adults aged 65+), and who had a history of care with one of the study clinics (at least 1 office visit within the 12 months prior to the observation period). Vaccination rates measured whether patients in FM and comparison IM clinics were up-to-date with pneumococcal vaccination according to ACIP guidelines in place at the end of each observation period. For at-risk adults age 19–64 years old, vaccination rates measured whether adults were up-to-date with PPSV23 vaccination by the end of the observation period. For high-risk adults age 19–64 years old, vaccination rates measured whether adults were up-to-date with the appropriate number of doses (PCV13, PPSV23 or both) by the end of the observation period.

**Table 1:** Interventions implemented during observation periods by clinic group: vaccination rate analyses

Observation period	Time Frame	Family Medicine Clinics (FM Group A)	Family Medicine Clinics (FM Group B)	Internal Medicine Clinics (IM Group C)
Baseline	August 1, 2013 - July 31, 2014	Current Practice	Current Practice	Current Practice
Interim	May 1, 2015 - April 30, 2016	Clinical workflow redesign	Current Practice	Current Practice
Follow-up	May 1, 2016 - Jul 31, 2017	Workflow redesign plus BPA tool & Health Maintenance Notification	Best practice alert tool & Health Maintenance Notification	Health Maintenance Notification

The baseline period (August 1, 2013 – July 31, 2014) represents the period of time prior to BPA, health maintenance notifications, and workflow improvements. The interim period (May 1, 2015 – April 30, 2016) represents the period of time during which 4 of the 11 FM Clinics undertook a redesign of pneumococcal vaccination workflow processes but before the BPA tool and health maintenance notifications were implemented. The follow-up period (May 1, 2016 – July 31, 2017) represents the time during which the BPA tool was rolled out to all FM Clinics, and the health maintenance notification rolled out to both FM and IM clinics.

Adherence to the 2-dose vaccination schedule in older adults was measured through a longitudinal analysis of immunocompetent adults aged 65+ who received the first pneumococcal vaccination between June 1, 2014 and April 30, 2016, and who were due to receive the second vaccination between December 1, 2014 and July 31, 2017. The proportion of patients who completed the series was compared by clinical decision support tool and workflow redesign status at the time the patient’s second dose was due: [1] current practice (no intervention); [2] workflow redesign only; [3] health maintenance notification only; [4] BPA and health maintenance notification; and [5] workflow redesign and BPA and health maintenance notification (Table 2).

**Table 2:** Interventions implemented at the time of first and second pneumococcal dose: series completion analysis

Clinic Group	Timeframe for Second Dose	Intervention	Exposure Group
June 2014–April 2016: Dose 1 timeframe			
B, C	June 2014–April 2016	Current practice	1
A	June 2014–April 2015	Current practice	1
A	May 2015–April 2016	Workflow redesign only	2
Dec 2014 - July 2017: Dose 2 timeframe			
B, C	May 2015–April 2016	Current practice	1
A	May 2015–April 2016	Workflow redesign only	2
C	May 2016–July 2017	Health maintenance only	3
B	May 2016–July 2017	BPA & health maintenance	4
A	May 2016–July 2017	BPA & health maintenance + Workflow redesign	5

### Statistical analysis

Descriptive statistics (count, percentage, mean, standard deviation, 95% confidence intervals) were used to compare patient characteristics between clinic groups for each observation period.

For each observation period (Baseline, Interim, and Follow-up), the vaccination rate was identified for the study clinic populations and was defined as:

$$ \frac{\mathrm{Number}\ \mathrm{of}\ \mathrm{patients}\ \mathrm{whose}\ \mathrm{vaccination}\ \mathrm{status}\ \mathrm{was}\ \mathrm{up}-\mathrm{to}-\mathrm{date}\ \mathrm{according}\ \mathrm{to}\ \mathrm{ACIP}\ \mathrm{guidelines}\ \mathrm{by}\ \mathrm{the}\ \mathrm{end}\ \mathrm{of}\ \mathrm{the}\ \mathrm{observation}\ \mathrm{period}}{\mathrm{Number}\ \mathrm{of}\ \mathrm{patients}\ \mathrm{recommended}\ \mathrm{for}\ \mathrm{pneumococcal}\ \mathrm{vaccination}\ \mathrm{according}\ \mathrm{to}\ \mathrm{ACIP}\ \mathrm{guidelines}\ \mathrm{by}\ \mathrm{the}\ \mathrm{end}\ \mathrm{of}\ \mathrm{the}\ \mathrm{observation}\ \mathrm{period}} $$

To assess vaccination rates while minimizing confounding from the non-random sample, the difference-in-differences (DD) method with a block bootstrap was used, with exposure defined as the intervention in place at the patient’s assigned clinic at the end of each observation period. The DD approach controls for unobserved differences between clinics that are related to both the intervention and vaccination rates, while controlling for trends over time that may affect vaccination rates across all clinics. [9, 10] Demographic and clinical covariates were included in the regression models to control for differences in the composition of patient groups that could lead to confounding.

Mixed effects logistic regression under the generalized linear mixed models (GLMMs) was used to identify associations between intervention exposure at the time the second dose was due and series completion in older adults, controlling for potential confounders. Mixed effects logistic regression accounts for the dependence of observations because of the clustering of patients within clinics. Due to the hierarchical structure of the data, three level mixed effects logistic regression was also performed to account for possible clustering both within clinics and time period.

## Results

### Vaccination rates in at-risk adults age 19–64 years

Of 18,851 individuals age 19–64 years who met study inclusion criteria in the baseline period, 16,193 (86%) were considered at-risk (immunocompetent with chronic medical conditions), with 84% (18,423 out of 21,872 patients) and 86% (21,498 out of 25,110 patients) of patients identified as at-risk in the interim and follow-up periods, respectively.

Age and sex of at-risk adults age 19–64 for each observation period are provided in Table 3. Characteristics of at-risk patients age 19–64 years are presented overall and by clinic for each observation period in the Additional file 1: Tables S2-S4. Overall, patient age, sex, and race were similar across the 3 time periods; patient mean age was 44 years, 55% of patients were female, and nearly three-quarters were Caucasian. The proportion of patients who were commercially insured ranged from 57% in the first observation period to 67% in the third observation period. Cigarette smoking (40 to 45%), chronic lung disease (33 to 38%), and diabetes (28 to 29%) were the 3 most common at-risk conditions across the observation periods.

**Table 3:** Demographic characteristics for adults populations studied, stratified by observation period

	At-risk adults aged 19–64 years			High-Risk Adults Age 19–64 Years			Immunocompetent adults aged 65+			Immunocompromised Adults Age 65+		
	Aug 2013 – Jul 2014 (Baseline Period) *N* = 16,193	May 2015 – Apr 2016 (Interim Period) *N* = 18,423	May 2016 – Jul 2017 (Follow-up Period) *N* = 21,498	Aug 2013 – Jul 2014 (Baseline Period) *N* = 2658	May 2015 – Apr 2016 (Interim Period) *N* = 3449	May 2016 – Jul 2017 (Follow-up period) *N* = 3612	Aug 2013 – Jul 2014 (Baseline Period) *N* = 9480	May 2015 – Apr 2016 (Interim Period) *N* = 11,318	May 2016 – Jul 2017 (Follow-up Period) *N* = 13,341	Aug 2013 – Jul 2014 (Baseline Period) *N* = 2577	May 2015 – Apr 2016 (Interim Period) *N* = 3200	May 2016 – Jul 2017 (Follow-up Period) *N* = 3348
Age												
Median age	45	45	45	53	53	53	72	71	71	74	74	73
Mean age (SD)	44.13 (12.70)	44.06 (12.73)	43.87 (12.79)	50.28 (10.99)	50.15 (11.08)	49.75 (11.26)	73.49 (7.19)	73.19 (7.06)	73.13 (7.02)	75.11 (7.61)	74.81 (7.30)	74.60 (7.21)
Sex n (%)												
Male	7327 (45.25)	8290 (45.00)	9733 (45.27)	1111 (41.80)	1411 (40.91)	1402 (38.82)	3698 (39.01)	4555 (40.25)	5353 (40.12)	1308 (50.76)	1596 (49.88)	1654 (49.40)
Female	8866 (54.75)	10,133 (55.00)	11,765 (54.73)	1547 (58.20)	2038 (59.09)	2210 (61.18)	5782 (60.99)	6763 (59.75)	7988 (59.88)	1269 (49.24)	1604 (50.13)	1694 (50.60)

In each observation period, patients in the IM Group C clinics were 4–5 years older on average than patients in FM A and B clinics. IM Group C clinics also had a lower proportion of female patients and cigarette smokers compared to FM A and B clinics, and a higher proportion of patients with diabetes. FM Group B clinics had a higher proportion of Hispanic patients and a lower proportion of commercially insured patients than FM Group A or IM Group C clinics in each observation period.

Vaccination rates in the at-risk 19–64 years old population measured whether adults were up-to-date with PPSV23 vaccination by the end of the observation period. The unadjusted vaccination rate in this group overall improved from 25.3% in the baseline period to 29.7 and 36.1% in the interim and follow up periods respectively (*P* < 0.001) (Additional file 2: Figure S1). While the vaccination rate improved in each clinic over the 3 observation periods, the rate was significantly lower in the intervention clinics (FM Group A and FM Group B) than the IM Group C clinics in the baseline and interim periods. No difference was detected during the follow-up period among the 3 clinics.

DD analyses showed that the clinics with the BPA, with or without workflow redesign (FM Group A and FM Group B), had greater improvement in vaccination rates from baseline to follow up relative to clinics with health maintenance notifications only (IM Group C clinics), controlling for demographic and clinical characteristics and underlying trends. Implementing both workflow redesign and the BPA (FM Group A) raised the vaccination rate by 8 percentage points (*P* < 0.001), and implementing the BPA only (FM Group B) raised the rate by 7 percentage points (P < 0.001), relative to implementing health maintenance notifications only (IM Group C) (Additional file 3: Figure S2).

### Vaccination rates in high-risk adults age 19–64 years

Of 18,851 patients age 19–64 years who met study inclusion criteria in the baseline period, 14% (2658 patients) were categorized as high-risk (immunocompromised) with 16% (3449 out of 25,110 patients) and 14% (3612 out of 25,110 patients) identified as high-risk during the interim and follow-up periods, respectively.

Age and sex of high-risk adults age 19–64 for each observation period are provided in Table 3. Characteristics of high-risk patients age 19–64 years are presented overall and by clinic for each observation period in the Additional file 1: Tables S5-S7. Overall, patient age, sex, race and insurance status were similar across the 3 time periods; patient mean age was 50 years, 58–61% of patients were female, 78–82% were Caucasian, and nearly two-thirds were commercially insured. Generalized malignancy (48 to 62%), iatrogenic immunosuppression (15 to 30%), and chronic renal failure (12 to 13%) were the 3 most common high-risk conditions across the observation periods.

In each observation period, patients in the IM Group C clinics were 2–3 years older on average than patients in FM A and B clinics. IM Group C clinics also had a higher proportion of patients with race classified as unknown, and a higher proportion of Medicare insured and chronic renal failure patients compared to FM A and B clinics. No significant differences in sex distribution were observed between clinics in the 3 observation periods.

Vaccination rates in the high-risk 19–64 years old population measured whether adults were up-to-date with the appropriate number of doses (PCV13, PPSV23 or both) by the end of the observation period. The unadjusted vaccination rate in high-risk patients 19–64 years old overall improved from 9.4% in the baseline period to 12.4 and 21.1% in the interim and follow up periods respectively (*P* < 0.001) (Additional file 4: Figure S3). While the vaccination rate improved in each clinic over the 3 observation periods, vaccination rates were significantly lower in the intervention clinics (FM Group A and FM Group B) than the IM Group C comparison clinics in all time periods. Rates improved notably for PCV13 in each clinic, increasing from approximately 2% in the baseline period to 20% or higher in the follow up period (Table 4).

**Table 4:** Vaccination rates for PCV13 and PPSV23 overall and by intervention group within the adult populations studied

Vaccination Rates	Family Med Clinics (A)				Family Med Clinics (B)				Internal Med Clinics (C)			
	Baseline	Interim	Follow-up	*p*-value	Baseline	Interim	Follow-up	p-value	Baseline	Interim	Follow-up	*p*-value
High risk adults aged 19–64 years												
PCV13 eligible	691	961	994		732	982	1088		1030	1369	1308	
Vaccinated (n)	14	117	199	**<.0001**	17	135	261	**<.0001**	30	247	363	**<.0001**
Vaccinated (%)	2.03	12.17	20.02		2.32	13.75	23.99		2.91	18.04	27.75	
PPSV23 eligible	734	1000	1048		785	1024	1139		1125	1395	1393	
Vaccinated (n)	146	278	302	**<.0001**	182	260	351	**0.0004**	317	427	491	**0.0005**
Vaccinated (%)	19.89	27.80	28.82		23.18	25.39	30.82		28.18	30.61	35.25	
Immunocompetent adults aged 65+												
PCV13 eligible		2429	2807			2966	3724			5403	5530	**<.0001**
Vaccinated (n)	NA	997	1677			971	2177			2842	3778	
Vaccinated (%)	NA	41.05	59.74			32.74	58.46			52.60	68.32	
PPSV23 eligible	2162	1796	2167		2519	2360	2683		4799	3519	4687	
Vaccinated (n)	1368	981	1219	**<.0001**	1356	1196	1301	**0.001**	3253	1999	3017	**<.0001**
Vaccinated (%)	63.27	54.62	56.25		53.83	50.68	48.49		67.78	56.81	64.37	
Immunocompromised Adults Aged 65+												
PCV13 eligible	453	578	600		452	619	684		1374	1878	1777	
Vaccinated (n)	2	248	416	**<.0001**	4	239	440	**<.0001**	9	1091	1323	**<.0001**
Vaccinated (%)	0.44	42.91	69.33		0.88	38.61	64.33		0.66	58.09	74.45	
PPSV23 eligible	516	589	637		512	642	742		1549	1894	1907	
Vaccinated (n)	369	391	434	0.180	327	416	492	0.6559	1144	1303	1397	**0.0011**
Vaccinated (%)	71.51	66.38	68.13		63.87	64.8	66.31		73.85	68.8	73.26	

Bold text indicates statistical significance

In DD analyses, controlling for demographic and clinical characteristics, implementing the BPA with or without workflow redesign did not significantly affect vaccination rates from baseline to follow up (difference relative to health maintenance notifications only in the IM Group C comparison clinics: − 2 percentage points, *P* = 0.365 for FM Group B; − 1 percentage point, *P* = 0.704 for FM Group A) (Additional file 5: Figure S4).

### Vaccination rates in immunocompetent adults aged 65+

Of 12,057 individuals aged 65+ who met study inclusion criteria during the baseline period, 9480 (79%) were considered immunocompetent, with 11,318 of 14,518 (78%) and 13,341 of 16,689 (80%) identified as immunocompetent in the interim and follow-up periods, respectively.

Age and sex of immunocompetent adults aged 65+ for each observation period are provided in Table 1. Characteristics of immunocompetent adults aged 65+ are presented overall and by clinic for each observation period in the Additional file 1: Tables S8-S10. Overall, patient age, sex, race and insurance status were similar across the 3 time periods; patient mean age was 73 years, 60% of patients were female, 80% were Caucasian, and 82% were insured by Medicare. Diabetes (25%), chronic heart disease (21%) and chronic lung disease (20%), were the 3 most common at-risk conditions across the observation periods.

In each observation period, patients in the IM Group C clinics were 1–2 years older on average than patients in FM A and B clinics. IM Group C clinics also had a higher proportion of Medicare-insured patients compared to FM A and B clinics, and a higher proportion of patients with chronic heart and lung disease. FM Group B clinics had a higher proportion of Hispanic patients than FM Group A or IM Group C clinics in each observation period.

Vaccination rates in immunocompetent adults aged 65+ measured whether adults were up-to-date with PPSV23 vaccination by the end of the baseline period, and whether adults were up-to-date with the appropriate number of doses (PCV13, PPSV23 or both) by the end of the interim and follow-up periods based on changes in ACIP pneumococcal guidelines during that time. The unadjusted vaccination rate overall across all clinics decreased from 63.1% in the baseline period to 43.6% in the interim period, but rebounded to 60.5% in the follow-up period (Additional file 6: Figure S5). All clinic groups saw a decrease in PPSV23 vaccination rates in the interim period, reflecting the guideline change, with significant increases in PCV13 vaccination during both the interim and follow-up periods (Table 4).

In DD analyses, controlling for demographic and clinical characteristics, clinics that implemented the BPA with workflow redesign (FM Group A) saw a modest decrease in the vaccination rates from baseline to follow-up relative to IM Group C clinics, but the reduction was not statistically significant (coef − 0.01; *P* = 0.664). Implementing the BPA (FM Group B) increased the vaccination rate 6 percentage points from baseline to follow-up (coef 0.06; *P* = 0.001) relative to the IM Group C clinics. (Additional file 7: Figure S6).

### Vaccination rates in immunocompromised adults aged 65+

Of 12,057 adults aged 65+ who met study inclusion criteria during the baseline period, 2577 (21%) were considered immunocompromised. As with the immunocompetent cohort aged 65+, the number of patients identified increased with each time period with 3200 (22%) included in the interim period and 3348 (20%) in the follow-up period.

Age and sex of immunocompromised adults aged 65+ for each observation period are provided in Table 1. Characteristics of immunocompromised adults age 65 years and older are presented overall and by clinic for each observation period in the Additional file 1: Tables S11-S13. Overall, patient age, sex, race and insurance status were similar across the 3 time periods; patient mean age was 75 years, 50% of patients were female, 86% were Caucasian, and 87% were insured by Medicare. Generalized malignancy (62–74%), chronic renal failure (14–16%), solid organ transplant (10–12%) and iatrogenic suppression (9–19%), were the 4 most common at-risk conditions across the observation periods.

In each observation period, patients in the IM Group C clinics were approximately 1 year older on average than patients in FM A and B clinics. FM Group B clinics had a higher proportion of Hispanic patients than FM Group A or IM Group C clinics. There were no significant differences in the most common immunosuppressive conditions between clinics in any of the 3 observation periods.

Vaccination rates in this population measured whether adults were up-to-date with the appropriate number of doses (PCV13, PPSV23 or both) by the end of the observation period. The unadjusted vaccination rate in high-risk patients aged 65+ improved from 12.0% in the baseline period to 40.9 and 60.0% in the interim and follow up periods respectively (*P* < 0.001) (Additional file 8: Figure S7). While there was no difference in the vaccination rate between clinics in the baseline period, vaccination rates in the intervention FM A and B clinics were significantly lower than in the IM Group C comparison clinics in the interim and follow up periods. Rates improved significantly for PCV13 in each clinic, increasing from < 1% in the baseline period to 64% or higher in the follow up period (Table 4).

In DD analyses, controlling for demographic and clinical characteristics, the adjusted vaccination rates improved substantially from baseline to follow-up in all clinic groups. However, the increase in vaccination rates for clinics implementing both the BPA and workflow redesign (FM Group A) was 6 percentage points less (coef − 0.06; *P* = 0.041) than in the IM Group C comparison clinics. Similarly, the increase in vaccination rates in clinics implementing only the BPA (FM Group B) was 11 percentage points lower (coef − 0.11; *P* < 0.001) than in the IM comparison clinics (Additional file 9: Figure S8).

### Series completion in adults aged 65+

A total of 1710 immunocompetent adults aged 65+, with documentation of a first pneumococcal vaccination with either PCV13 or PPSV23 during the baseline period, were included in the analysis of series completion. Of these, 516 patients (30.2%) were administered the second pneumococcal dose within 6–15 months after the first dose. Series completion was examined by the presence of workflow redesign and/or clinical decision support tools in place at the time the second dose was due. The unadjusted proportion of patients who completed the series ranged from 17% in patients who received the second dose in clinics who had undertaken workflow redesign but before clinical decision support tools were implemented, to 35.2 and 35.6% in clinics with health maintenance notifications with and without the BPA, respectively (*P* < 0.001) (Additional file 10: Figure S9).

In adjusted analyses, patients attending a clinic that implemented the BPA plus health maintenance notifications and workflow redesign (all 3 interventions) had 1.55 times higher odds (Odds ratio (OR) 1.55; *P* = 0.014, 95% CI 1.09, 2.19) to receive the second pneumococcal dose than patients attending a usual practice clinic (i.e., no intervention) (Table 5). Patients attending a clinic that had implemented both the BPA and health maintenance reminders (OR 1.48; *P* = 0.019, 95% CI 1.07, 2.05), or just the health maintenance reminders (OR 1.83; *P* < 0.0001, 95% CI 1.39, 2.40) were also more likely to receive the second vaccination to complete the series. Patients attending clinics that undertook workflow redesign but before implementation of the BPA or health maintenance tools were less likely to have completed the series compared with patients attending a usual practice clinic (OR 0.53; *P* = 0.005, 95% CI 0.34, 0.82). Results from the tree level mixed effects logistics regression were consistent with the results from the two level regression.

**Table 5:** Likelihood of Completing the Pneumococcal Vaccination Series by Interventions in Immunocompetent Adults aged 65+ (Mixed effects logistic regression)

	OR	*p*-value	95% Conf. Interval	
Intervention				
Current practice (ref)				
HM Only	1.83	**<.0001**	1.39	2.40
Revised BPA + HM	1.48	**0.019**	1.07	2.05
Workflow redesign only	0.53	**0.005**	0.34	0.82
Revised BPA + HM + Workflow redesign	1.55	**0.014**	1.09	2.19
Age at time of first dose (ref 65 years)				
66–75	0.57	**<.0001**	0.44	0.73
> 75	0.34	**<.0001**	0.24	0.48
Gender (ref male)				
Female	1.06	0.578	0.85	1.33
Insurance Status (ref Medicare)				
Medicaid	1.27	0.642	0.46	3.51
Commercial	1.43	**0.007**	1.10	1.85
Uninsured	1.29	0.481	0.64	2.58
Other	2.07	0.617	0.12	36.07
Charlson Comorbidity Index	1.00	0.914	0.94	1.08
Chronic heart disease	0.64	**0.011**	0.45	0.90
Pre-index factors				
Prior Pneumonia-Related Diagnosis	0.97	0.894	0.63	1.50
Number of office visits 12-mos pre-index	1.02	0.345	0.98	1.07

Bold text indicates statistical significance

## Discussion

This vaccination quality improvement study assessed the association between implementing a pneumococcal BPA tool reflecting current guidelines with and without workflow redesign on pneumococcal vaccination rates in primary care clinics affiliated with an academic medical center. The study did not consistently find a positive association between the implementation of this tool and vaccination rates in a primary care setting in each of the adult cohorts studied. Of the four groups for whom pneumococcal vaccination is recommended, use of a BPA were associated with improved vaccination rates in at-risk adults aged 19–64 years and immunocompromised adults aged 65+ when compared with clinics who implemented health maintenance notifications only.

In at-risk adults aged 19–64 years, clinics implementing the BPA with or without workflow redesign had a greater improvement in pneumococcal vaccination rates than comparison clinics who implemented health maintenance notifications only. No difference was observed in the age 19–64 high-risk group between clinics with the BPA or workflow redesign versus the comparison clinics. Similarly, in the aged 65+ immunocompetent cohort, no difference was seen between clinics that implemented both the BPA and workflow redesign versus comparison clinics. However, clinics that implemented just the BPA (FM Group B) had a greater improvement in vaccination rate (coef 0.06; *P* = 0.001) relative to the IM Group C clinics.

In immunocompromised adults aged 65+, clinics that implemented the BPA with or without workflow redesign experienced a smaller improvement in vaccination rates relative to the comparison clinics. However, over the study period there was a 4-fold improvement in vaccination rates for all clinic groups (e.g. from 9 to 10% at baseline to 46–50% at follow-up), driven by a substantial uptake of PCV13 vaccination.

The second objective of the study was to longitudinally assess series completion over time in a cohort of immunocompetent older adults. This analysis found that patients attending clinics where the clinical decision support tools were implemented at the time their second dose was due, including BPA with health maintenance or health maintenance notifications alone, were more likely to receive the second vaccination than patients attending clinics without clinical decision support tools. Patients in the clinics that had undertaken workflow changes but before implementing the BPA and health maintenance tools were less likely to have received the second dose than those in usual care clinics. This finding was unexpected given the clinics’ focus on improving pneumococcal vaccination rates, but may reflect challenges of implementing new workflows during the period following guideline updates for this population.

Clinical decision support tools can help health care providers identify patients who are eligible for vaccinations. [6] For instance, the impact of an electronic medical record-based BPA that alerted providers to administer influenza and pneumococcal vaccines to adult patients taking immunosuppressive drugs was evaluated at the Geisinger Health System Rheumatology Department. Following BPA implementation, the pneumococcal vaccination rate in immunocompromised adults doubled from 19 to 41% (*p* < 0.001). [7]

On the other hand, numerous barriers to clinical decision support implementation have been also identified, which may help explain the mixed findings in this study. Barriers include competing clinical demands, reliability of information, complexity, physician attitudes, too many unwanted alerts, poor alert timing, and lack of acceptance of CDS tools. [8, 11, 12]

While U of U Health clinicians voiced a need for assistance with implementing pneumococcal vaccination guidelines, the guidelines and the supporting logic are complex. Even with extensive input from clinicians and implementation oversight by the U of U Health clinical decision support committee, it was challenging to systematically and accurately capture conditions that make patients candidates for pneumococcal vaccination in the BPA. This was particularly true for patients immunosuppressed at the time of the analyses and those with chronic lung disease or heart disease. While the U of U Health clinical decision support development team was successful in integrating a two-way, real-time information exchange with the state immunization registry to help improve accuracy and completeness of vaccination history data, numerous reports of actual or perceived “misfires” were made by clinicians after implementation, which may have reduced provider confidence in the information.

A positive association between the BPA in at-risk adults age 19–64 years and series completion in immunocompetent adults aged 65+ was identified. In these scenarios, providers only needed to provide a single PPSV-23 dose to at-risk adults age 19–64 and provide the second dose in the recommended series for adults aged 65+. In a post-hoc analysis, vaccination rates were further explored by select comorbidities. Patients with diabetes had higher vaccination rates and greater improvement from baseline to follow-up in the clinics with the BPA (FM Group A & B) versus comparison clinics (IM Group C). This same pattern was not observed for chronic lung and heart disease. This observed pattern may be due to greater complexity in interpreting which patients fit the definition of at-risk for heart and lung disease than for diabetes. Overall, these findings suggest that the BPA was associated with an increase in pneumococcal vaccination rates when applying the guidelines in less complex patient care scenarios.

Much of the gain in pneumococcal vaccination rates in high-risk adults age 19–64 years and immunocompromised adults aged 65+ was due to the large uptake in PCV13 vaccinations. While no system-wide efforts were in place to increase PCV13 vaccinations at U of U Health,this gain corresponds with the timing of guideline changes that added PCV13 to the aged 65+ immunocompetent patient recommendations. This guideline update may have universally increased consumer and provider awareness and overshadowed the effect of the BPA and workflow redesign on vaccination rates for all age and risk groups for which PCV-13 is recommended, even though recommendations for sequential PCV13-PPSV23 vaccination have been in place for high-risk/immunocompromised adults age 19 and above since 2012.

Finally, health maintenance reminders were implemented system-wide at U of U Health in the follow-up period, including in the IM Group C comparison clinics. Based on other, recent experience at U of U Health, it is possible that health maintenance reminders may be better received and more likely to be followed than BPAs. This study was not specifically designed to answer that question; however, it will be the topic of future research.

### Limitations

This study has a number of limitations that could affect results. First, this was an observational study of a planned quality improvement initiative, and thus clinics were not randomized to interventions. While an internal comparison group was included and a statistical method employed to help control for underlying trends, there may be other factors that affected vaccination rates which were not controlled for.

Vaccination status was based upon documentation of vaccination administration and patient reported history in the electronic medical record by providers/clinic staff. While documentation of U of U Health administered vaccines should be complete, patient recall of pneumococcal vaccination given outside U of U Health may be inaccurate. Access to USIIS data should help to reduce recall bias, however documentation in USIIS is only mandatory for adult vaccinations administered in pharmacies in Utah. Thus, this lack of complete and accurate vaccination history in U of U Health systems may have introduced misclassification of pneumococcal vaccination status leading to an overall underestimation of vaccination rates.

Vaccination rates were calculated using the total number of eligible adults age 19–64 years who attended clinics, rather than the total eligible population within the catchment area of each clinic group. Whilst the denominator could be the total population within a defined geographical area, these data were not collected as part of this study.

ICD-9/10 codes were used to identify vaccine eligible conditions. While the presence of 2 codes for a given condition on separate days was required to avoid misclassifying patients, diagnosis codes often lack the specificity to accurately identify at-risk, immunocompetent versus high-risk and currently immunocompromised cohorts. To gain provider confidence, the BPA was turned off for approximately 2 weeks during the first month of implementation while adjustments were made to at-risk/high-risk condition groupers and clinical decision support logic rules. Thus, reporting of vaccine eligible conditions reflects these modifications over time. Since the time the BPA was turned off represents only a small portion of the total follow-up time, it is unlikely that vaccination rates or series completion was significantly affected.

Finally, patient clinic assignment was based on the location of the patient’s last office visit during the observation period for the analysis of vaccination rates and at the time the second dose was due for the series completion evaluation. Patients in the U of U Health tend to visit the same clinic on an ongoing basis, but the assigned clinic may not have been the patient’s primary clinic leading to misclassification of exposure. Since the same method for assigning clinic was used for all patients, it is unlikely that this bias favors one clinic group over the other.

## Conclusion

A pneumococcal BPA tool that reflects current guidelines implemented with and without workflow redesign improved vaccination rates for at-risk adults age 19–64 years and increased the likelihood of adults aged 65+ receiving their second dose to complete the recommended 2-dose series. However, in other adult patient groups, the BPA was not consistently associated with improvements in pneumococcal vaccination rates. Additional research is warranted to identify barriers to acting upon the BPA and to assess the impact of health maintenance reminders as an alternative approach to promoting adherence to pneumococcal vaccination guidelines.

## Additional files

Additional file 1: **Table S1.** Recommended immunization and intervals, by risk and age groups, for persons with indications to receive pneumococcal immunization. **Table S2.** Demographic Characteristics for At-Risk Adults Age 19–64 Years Overall and Stratified by Intervention Group - Aug 2013 - Jul 2014, Baseline Period. **Table S3.** Demographic Characteristics for At-Risk Adults Age 19–64 Years Overall and Stratified by Intervention Group - May 2015 - Apr 2016 (Interim Period). **Table S4.** Demographic Characteristics for At-Risk Adults Age 19–64 Years Overall and Stratified by Intervention Group - May 2016 - Jul 2017 (Follow-up Period). **Table S5.** Demographic Characteristics for High-Risk Adults Age 19–64 Years Overall and Stratified by Intervention Group - Aug 2013 - Jul 2014 (Baseline Period). **Table S6.** Demographic Characteristics for High-Risk Adults Age 19–64 Years Overall and Stratified by Intervention Group - May 2015 - Apr 2016 (Interim Period). **Table S7.** Demographic Characteristics for High-Risk Adults Age 19–64 Years Overall and Stratified by Intervention Group - May 2016 - Jul 2017 (Follow-up Period). **Table S8.** Demographic Characteristics for Immunocompetent Adults Aged 65+ Overall and Stratified by Intervention Group - Aug 2013 - Jul 2014 (Baseline Period). **Table S9.** Demographic Characteristics for Immunocompetent Adults Aged 65+ Overall and Stratified by Intervention Group - May 2015 - Apr 2016 (Interim Period). **Table S10.** Demographic Characteristics for Immunocompetent Adults Aged 65+ Overall and Stratified by Intervention Group - May 2016 - Jul 2017 (Follow-up Period). **Table S11.** Demographic Characteristics for Immunocompromised Adults Aged 65+ Overall and Stratified by Intervention Group - Aug 2013 - Jul 2014 (Baseline Period). **Table S12.** Demographic Characteristics for Immunocompromised Adults Aged 65+ Overall and Stratified by Intervention Group - May 2015 - Apr 2016 (Interim Period). **Table S13.** Demographic Characteristics for Immunocompromised Adults Aged 65+ Overall and Stratified by Intervention Group - May 2016 - Jul 2017 (Follow-up Period). (DOCX 132 kb)

Additional file 2: **Figure S1.** Vaccination Rates for At-Risk Adults Age 19–64 Years by Clinic Group and Overall. Description: The vaccination rates of at-risk adults age 19–64 years by clinic group and overall over the three time periods studied. (DOCX 59 kb)

Additional file 3: **Figure S2.** Intervention Effect on Pneumococcal Vaccination Rates: Implementing BPA and/or Workflow Redesign versus Health Maintenance Notifications – At-Risk Patients age 19–64 Years. Description: Pneumococcal vaccination rates in at-risk patients age 19–64 years in clinics implementing BPA and/or workflow redesigns, versus health maintenance notifications. (DOCX 31 kb)

Additional file 4: **Figure S3.** Vaccination Rates for High-Risk Adults Age 19–64 Years by Clinic Group and Overall. Description: The vaccination rates of high-risk adults age 19–64 years by clinic group and overall over the three time periods studied. (DOCX 60 kb)

Additional file 5: **Figure S4.** Intervention Effect on Pneumococcal Vaccination Rates: Implementing BPA and/or Workflow Redesign versus Health Maintenance Notifications – High-Risk Patients age 19–64 Years. Description: Pneumococcal vaccination rates in high-risk patients age 19–64 years in clinics implementing BPA and/or workflow redesigns, versus health maintenance notifications. (DOCX 30 kb)

Additional file 6: **Figure S5.** Vaccination Rates for Immunocompetent Adults Aged 65+ by Clinic Group and Overall. Description: The vaccination rates of immunocompetent adults age 65+ years by clinic group and overall over the three time periods studied. (DOCX 54 kb)

Additional file 7: **Figure S6.** Intervention Effect on Pneumococcal Vaccination Rates: Implementing Workflow Redesign and/or BPA Versus Comparison Clinics based on Difference in Difference Analyses - Immunocompetent Patients aged 65+. Description: Pneumococcal vaccination rates in immunocompetent patients age 65+ years in clinics implementing workflow redesign and/or BPA, versus comparison clinics based on difference in difference analyses. (DOCX 31 kb)

Additional file 8: **Figure S7.** Vaccination Rates for Immunocompromised Adults Aged 65+ by Clinic Group and Overall. Description: The vaccination rates of immunocompromised adults age 65+ years by clinic group and overall over the three time periods studied. (DOCX 64 kb)

Additional file 9: **Figure S8.** Intervention Effect on Pneumococcal Vaccination Rates: Implementing Workflow Redesign and/or BPA Versus Comparison Clinics based on Difference in Difference Analyses – Immunocompromised Patients aged 65+. Description: Pneumococcal vaccination rates in immunocompromised patients aged 65+ years in clinics implementing workflow redesign and/or BPA, versus comparison clinics based on difference in difference analyses. (DOCX 31 kb)

Additional file 10: **Figure S9.** Pneumococcal Vaccination Series Completion by Intervention Category. Description: Pneumococcal vaccination series completion by intervention category, including no intervention, and all three interventions overall. (DOCX 59 kb)

## Abbreviations

ACIP: Advisory Committee on Immunization Practices
BPA: Best Practice Alert
CPT: Care delivery and billing codes
DD: Difference in differences
EDW: Enterprise Data Warehouse
FM: Family Medicine
HIV: Human immunodeficiency virus
IM: Internal Medicine
PCV13: Pneumococcal conjugate vaccine
PPSV23: Pneumococcal polysaccharide vaccine
U of U Health: University of Utah Health
US: United States
USIIS: Utah State Immunization Information System
