# Supplementary material for: Impact of a guideline-based best practice alert on pneumococcal vaccination rates in adults in a primary care setting
Source: BMC Health Serv Res. 2019 Jul 10;19:474. doi: 10.1186/s12913-019-4263-2 (PMC6621991; doi:10.1186/s12913-019-4263-2)
Supplement: Supplementary file 1 — Table S1. Recommended immunization and intervals, by risk and age groups, for persons with indications to receive pneumococcal immunization. Table S2. Demographic Characteristics for At-Risk Adults Age 19–64 Years Overall and Stratified by Intervention Group - Aug 2013 - Jul 2014, Baseline Period. Table S3. Demographic Characteristics for At-Risk Adults Age 19–64 Years Overall and Stratified by Intervention Group - May 2015 - Apr 2016 (Interim Period). Table S4. Demographic Characteristics for At-Risk Adults Age 19–64 Years Overall and Stratified by Intervention Group - May 2016 - Jul 2017 (Follow-up Period). Table S5. Demographic Characteristics for High-Risk Adults Age 19–64 Years Overall and Stratified by Intervention Group - Aug 2013 - Jul 2014 (Baseline Period). Table S6. Demographic Characteristics for High-Risk Adults Age 19–64 Years Overall and Stratified by Intervention Group - May 2015 - Apr 2016 (Interim Period). Table S7. Demographic Characteristics for High-Risk Adults Age 19–64 Years Overall and Stratified by Intervention Group - May 2016 - Jul 2017 (Follow-up Period). Table S8. Demographic Characteristics for Immunocompetent Adults Aged 65+ Overall and Stratified by Intervention Group - Aug 2013 - Jul 2014 (Baseline Period). Table S9. Demographic Characteristics for Immunocompetent Adults Aged 65+ Overall and Stratified by Intervention Group - May 2015 - Apr 2016 (Interim Period). Table S10. Demographic Characteristics for Immunocompetent Adults Aged 65+ Overall and Stratified by Intervention Group - May 2016 - Jul 2017 (Follow-up Period). Table S11. Demographic Characteristics for Immunocompromised Adults Aged 65+ Overall and Stratified by Intervention Group - Aug 2013 - Jul 2014 (Baseline Period). Table S12. Demographic Characteristics for Immunocompromised Adults Aged 65+ Overall and Stratified by Intervention Group - May 2015 - Apr 2016 (Interim Period). Table S13. Demographic Characteristics for Immunocompromised Adults Aged 65+ Overall and Strat [file 12913_2019_4263_MOESM1_ESM.docx]

Additional file 1

| **Table S1. Recommended immunization and intervals, by risk and age groups, for persons with indications to receive pneumococcal immunization*** | | | | | | |
| --- | --- | --- | --- | --- | --- | --- |
|  | **PPSV23 Only** | | **Intervals for PCV13–PPSV23 sequence** | | **Intervals for PPSV23–PCV13 sequence** | |
| **Risk group/Underlying medical condition** | **19–64 years** | **≥65 years** | **19–64 years** | **≥65 years** | **19–64 years** | **≥65 years** |
| **No underlying chronic conditions** | 1 time | NA | NA | ≥1 year | NA | ≥1 year |
| **Immunocompetent persons:** chronic heart disease, chronic lung disease, diabetes mellitus, alcoholism, chronic liver disease/cirrhosis, cigarette smoking | NA | NA | NA | ≥1 year | NA | ≥1 year |
| **Immunocompetent persons:** cerebrospinal fluid leak, cochlear implant | NA | NA | ≥8 weeks | ≥8 weeks | ≥1 year | ≥1 year |
| **Persons with functional or anatomic asplenia:** sickle cell disease/other hemaglobinopathy, congenital or acquired asplenia | NA | NA | ≥8 weeks | ≥8 weeks | ≥1 year | ≥1 year |
| **Immunocompromised persons:** congenital or acquired immunodeficiency, human immunodeficiency virus infection, chronic renal failure, nephrotic syndrome, leukemia, lymphoma, Hodgkin disease, generalized malignancy, iatrogenic immunosuppression, solid organ transplant, multiple myeloma | NA | NA | ≥8 weeks | ≥8 weeks | ≥1 year | ≥1 year |

*Based on Advisory Committee on Immunization Practices (ACIP) Guidelines, United States, September 2015. Based on Kobayashi M, Bennett NM. MMWR. September 4, 2015. 64 (34): 944-947

| **Table S2. Demographic Characteristics for At-Risk Adults Age 19-64 Years Overall and Stratified by Intervention Group – Aug 2013 – Jul 2014** | | | | | | | |
| --- | --- | --- | --- | --- | --- | --- | --- |
| **Demographic Characteristics for At-Risk Adults 19-64 Years (Baseline Period)** | **Total N=16,193** | **Family Med Clinics (A) N=5,177** | **Family Med Clinics (B) N=5,806** | **Internal Med Clinics (C) N=5,210** | **p-value AvB*** | **p-value AvC*** | **p-value BvC*** |
| **Median age (years)** | 45 | 43 | 43 | 50 | **0.0172** | **<0.0001** | **<0.0001** |
| Mean age (SD) | 44.13 (12.70) | 43.10 (12.58) | 42.52 (12.76) | 46.96 (12.26) |  |  |  |
| **Gender n (%)** |  |  |  |  | 0.0727 | **0.0228** | **<0.0001** |
| Male | 7327 (45.25) | 2337 (45.14) | 2522 (43.44) | 2468 (47.37) |  |  |  |
| Female | 8866 (54.75) | 2840 (54.86) | 3284 (56.56) | 2742 (52.63) |  |  |  |
| **Race n (%)** |  |  |  |  | **<0.0001** | **<0.0001** | **<0.0001** |
| Caucasian | 11922 (73.62) | 4074 (78.69) | 4067 (70.05) | 3781 (72.57) |  |  |  |
| Black | 453 (2.80) | 115 (2.22) | 166 (2.86) | 172 (3.30) |  |  |  |
| Hispanic | 2129 (13.15) | 509 (9.83) | 962 (16.57) | 658 (12.63) |  |  |  |
| Other | 1356 (8.37) | 403 (7.78) | 441 (7.60) | 512 (9.83) |  |  |  |
| Unknown | 333 (2.06) | 76 (1.47) | 170 (2.93) | 87 (1.67) |  |  |  |
| **Insurance status n (%)** |  |  |  |  | **<0.0001** | **<0.0001** | **<0.0001** |
| Medicare | 2008 (12.40) | 615 (11.88) | 585 (10.08) | 808 (15.51) |  |  |  |
| Medicaid | 3116 (19.24) | 971 (18.76) | 1066 (18.36) | 1079 (20.71) |  |  |  |
| Commercial | 9221 (56.94) | 3200 (61.81) | 3142 (54.12) | 2879 (55.26) |  |  |  |
| Uninsured | 1752 (10.82) | 371 (7.17) | 979 (16.86) | 402 (7.72) |  |  |  |
| Other | 95 (0.59) | 20 (0.39) | 33 (0.57) | 42 (0.81) |  |  |  |
| Unknown | 1 (0.01) | 0 (0.00) | 1 (0.02) | 1 (0.02) |  |  |  |
| **Comorbidities n (%)** |  |  |  |  |  |  |  |
| Chronic heart disease | 1584 (9.78) | 427 (8.25) | 447 (7.70) | 710 (13.63) | 0.2885 | **<0.0001** | **<0.0001** |
| Chronic lung disease | 5395 (33.32) | 1698 (32.80) | 1973 (33.98) | 1724 (33.09) | 0.1895 | 0.7521 | 0.3223 |
| Diabetes mellitus | 4474 (27.63) | 1324 (25.57) | 1500 (25.84) | 1650 (31.67) | 0.755 | **<0.0001** | **<0.0001** |
| Alcoholism | 32 (0.20) | 16 (0.31) | 8 (0.14) | 8 (0.15) | 0.055 | 0.0988 | 0.8283 |
| Chronic liver disease, cirrhosis | 1700 (10.50) | 482 (9.31) | 540 (9.30) | 678 (13.01) | 0.9861 | **<0.0001** | **<0.0001** |
| Cigarette smoking | 7295 (45.05) | 2473 (47.77) | 2643 (45.52) | 2179 (41.82) | **0.0184** | **<0.0001** | **<0.0001** |
| Mean Charlson Comorbidity Index (SD) | 1.37 (1.53) | 1.26 (1.44) | 1.25 (1.40) | 1.62 (1.72) | 0.6699 | **<0.0001** | **<0.0001** |
| *Comparisons between study clinics (e.g. A vs B) were made with Chi-square tests or ANOVA, as appropriate. | | | | | | | |

| **Table S3. Demographic Characteristics for At-Risk Adults Age 19-64 Years Overall and Stratified by Intervention Group – May 2015 – Apr 2016** | | | | | | | |
| --- | --- | --- | --- | --- | --- | --- | --- |
| **Demographic Characteristics for At-Risk Adults 19-64 Years (Interim Period)** | **Total N=18,423** | **Family Med Clinics (A) N=5,898** | **Family Med Clinics (B) N=7,015** | **Internal Med Clinics (C) N=5,510** | **p-value AvB*** | **p-value AvC*** | **p-value BvC*** |
| **Median age (years)** | 45 | 43 | 43 | 50 | 0.1272 | **<0.0001** | **<0.0001** |
| Mean age (SD) | 44.06 (12.73) | 42.78 (12.88) | 42.44 (12.52) | 47.49 (12.17) |  |  |  |
| **Gender n (%)** |  |  |  |  | 0.3441 | **<0.0001** | **<0.0001** |
| Male | 8290 (45.00) | 2604 (44.15) | 3039 (43.32) | 2647 (48.04) |  |  |  |
| Female | 10133 (55.00) | 3294 (55.85) | 3976 (56.68) | 2863 (51.96) |  |  |  |
| **Race n (%)** |  |  |  |  | **<0.0001** | **<0.0001** | **<0.0001** |
| Caucasian | 13460 (73.06) | 4597 (77.94) | 4857 (69.24) | 4006 (72.70) |  |  |  |
| Black | 504 (2.74) | 121 (2.05) | 211 (3.01) | 172 (3.12) |  |  |  |
| Hispanic | 2464 (13.37) | 598 (10.14) | 1178 (16.79) | 688 (12.49) |  |  |  |
| Other | 1659 (9.01) | 489 (8.29) | 614 (8.75) | 556 (10.09) |  |  |  |
| Unknown | 336 (1.82) | 93 (1.58) | 155 (2.21) | 88 (1.60) |  |  |  |
| **Insurance status n (%)** |  |  |  |  | **<0.0001** | **<0.0001** | **<0.0001** |
| Medicare | 2050 (11.13) | 647 (10.97) | 621 (8.85) | 782 (14.19) |  |  |  |
| Medicaid | 3125 (16.96) | 943 (15.99) | 1234 (17.59) | 948 (17.21) |  |  |  |
| Commercial | 11506 (62.45) | 3980 (67.48) | 4041 (57.61) | 3485 (63.25) |  |  |  |
| Uninsured | 1662 (9.02) | 312 (5.29) | 1080 (15.40) | 270 (4.90) |  |  |  |
| Other | 79 (0.43) | 15 (0.25) | 39 (0.56) | 25 (0.45) |  |  |  |
| Unknown | 1 (0.01) | 1 (0.02) | 0 (0.00) | 0 (0.00) |  |  |  |
| **Comorbidities n (%)** |  |  |  |  |  |  |  |
| Chronic heart disease | 1874 (10.17) | 493 (8.36) | 559 (7.97) | 822 (14.92) | 0.4195 | **<0.0001** | **<0.0001** |
| Chronic lung disease | 6561 (35.61) | 2112 (35.81) | 2474 (35.27) | 1975 (35.84) | 0.5219 | 0.9688 | 0.5033 |
| Diabetes mellitus | 5207 (28.26) | 1501 (25.45) | 1863 (26.56) | 1843 (33.45) | 0.153 | **<0.0001** | **<0.0001** |
| Alcoholism | 46 (0.25) | 15 (0.25) | 14 (0.20) | 17 (0.31) | 0.5127 | 0.5844 | 0.2232 |
| Chronic liver disease, cirrhosis | 1956 (10.62) | 524 (8.88) | 666 (9.49) | 766 (13.90) | 0.2329 | **<0.0001** | **<0.0001** |
| Cigarette smoking | 7822 (42.46) | 2702 (45.81) | 3076 (43.85) | 2044 (37.10) | **0.0254** | **<0.0001** | **<0.0001** |
| Other | 133 (0.72) | 47 (0.80) | 53 (0.76) | 33 (0.60) | 0.7894 | 0.2054 | 0.2921 |
| **Median Charlson Comorbidity Index** | 1 | 1 | 1 | 1 |  |  |  |
| Mean Charlson Comorbidity Index (SD) | 1.44 (1.58) | 1.30 (1.49) | 1.30 (1.48) | 1.76 (1.76) | 0.863 | **<0.0001** | **<0.0001** |
| *Comparisons between study clinics (e.g. A vs B) were made with Chi-square tests or ANOVA, as appropriate. | | | | | | | |

| **Table S4. Demographic Characteristics for At-Risk Adults Age 19-64 Years Overall and Stratified by Intervention Group – May 2016 – Jul 2017** | | | | | | | |
| --- | --- | --- | --- | --- | --- | --- | --- |
| **Demographic Characteristics for At-Risk Adults 19-64 Years (Follow-up Period)** | **Total N=21,498** | **Family Med Clinics (A) N=7,030** | **Family Med Clinics (B) N=8,517** | **Internal Med Clinics (C) N=5,951** | **p-value AvB*** | **p-value AvC*** | **p-value BvC*** |
| **Median age (years)** | 45 | 43 | 43 | 50 | 0.8815 | **<0.0001** | **<0.0001** |
| Mean age (SD) | 43.87 (12.79) | 42.59 (12.79) | 42.63 (12.68) | 47.17 (12.37) |  |  |  |
| **Gender n (%)** |  |  |  |  | 0.4558 | **0.0002** | **0.0013** |
| Male | 9733 (45.27) | 3102 (44.13) | 3809 (44.72) | 2822 (47.42) |  |  |  |
| Female | 11765 (54.73) | 3928 (55.87) | 4708 (55.28) | 3129 (52.58) |  |  |  |
| **Race n (%)** |  |  |  |  | **<0.0001** | **<0.0001** | **<0.0001** |
| Caucasian | 15597 (72.55) | 5374 (76.44) | 5979 (70.20) | 4244 (71.32) |  |  |  |
| Black | 593 (2.76) | 145 (2.06) | 235 (2.76) | 213 (3.58) |  |  |  |
| Hispanic | 2950 (13.72) | 785 (11.17) | 1387 (16.29) | 778 (13.07) |  |  |  |
| Other | 1985 (9.23) | 620 (8.82) | 734 (8.62) | 631 (10.60) |  |  |  |
| Unknown | 373 (1.74) | 106 (1.51) | 182 (2.14) | 85 (1.43) |  |  |  |
| **Insurance status n (%)** |  |  |  |  | **<0.0001** | **<0.0001** | **<0.0001** |
| Medicare | 2156 (10.03) | 677 (9.63) | 722 (8.48) | 757 (12.72) |  |  |  |
| Medicaid | 3023 (14.06) | 938 (13.34) | 1209 (14.20) | 876 (14.72) |  |  |  |
| Commercial | 14447 (67.20) | 4998 (71.10) | 5434 (63.80) | 4015 (67.47) |  |  |  |
| Uninsured | 1709 (7.95) | 362 (5.15) | 1089 (12.79) | 258 (4.34) |  |  |  |
| Other | 70 (0.33) | 27 (0.38) | 29 (0.34) | 14 (0.24) |  |  |  |
| Unknown | 93 (0.43) | 28 (0.40) | 34 (0.40) | 34 (0.40) |  |  |  |
| **Comorbidities n (%)** |  |  |  |  |  |  |  |
| Chronic heart disease | 2080 (9.68) | 585 (8.32) | 643 (7.55) | 852 (14.32) | 0.0757 | **<0.0001** | **<0.0001** |
| Chronic lung disease | 8208 (38.18) | 2690 (38.26) | 3254 (38.21) | 2264 (38.04) | 0.9403 | 0.7966 | 0.8436 |
| Diabetes mellitus | 6149 (28.60) | 1810 (25.75) | 2346 (27.54) | 1993 (33.49) | **0.0117** | **<0.0001** | **<0.0001** |
| Alcoholism | 56 (0.26) | 21 (0.30) | 16 (0.19) | 19 (0.32) | 0.158 | 0.8333 | 0.1133 |
| Chronic liver disease, cirrhosis | 2359 (10.97) | 652 (9.27) | 848 (9.96) | 859 (14.43) | 0.1517 | **<0.0001** | **<0.0001** |
| Cigarette smoking | 8595 (39.98) | 3039 (43.23) | 3460 (40.62) | 2096 (35.22) | **0.001** | **<0.0001** | **<0.0001** |
| Other | 120 (0.56) | 41 (0.58) | 45 (0.53) | 34 (0.57) | 0.6462 | 0.9291 | 0.73 |
| **Median Charlson Comorbidity Index** | 1 | 1 | 1 | 1 |  |  |  |
| Mean Charlson Comorbidity Index (SD) | 1.46 (1.60) | 1.34 (1.51) | 1.34 (1.46) | 1.78 (1.82) | 0.9279 | **<0.0001** | **<0.0001** |
| *Comparisons between study clinics (e.g. A vs B) were made with Chi-square tests or ANOVA, as appropriate. | | | | | | | |

| **Table S5. Demographic Characteristics for High-Risk Adults Age 19-64 Years Overall and Stratified by Intervention Group – Aug 2013 – Jul 2014** | | | | | | | |
| --- | --- | --- | --- | --- | --- | --- | --- |
| **Demographic Characteristics for High-Risk Adults 19-64 Years (Baseline Period)** | **Total N=2,658** | **Family Med Clinics (A) N=735** | **Family Med Clinics (B) N=789** | **Internal Med Clinics (C) N=1,134** | **p-value AvB*** | **p-value AvC*** | **p-value BvC*** |
| **Median age (years)** | 53 | 52 | 51 | 55 |  |  |  |
| Mean age (SD) | 50.28 (10.99) | 49.16 (11.42) | 49.08 (11.09) | 51.83 (10.44) | 0.8963 | **<0.0001** | **<0.0001** |
| **Gender n (%)** |  |  |  |  | 0.0858 | **0.0373** | 0.8222 |
| Male | 1111 (41.80) | 332 (45.17) | 322 (40.81) | 457 (40.30) |  |  |  |
| Female | 1547 (58.20) | 403 (54.83) | 467 (59.19) | 677 (59.70) |  |  |  |
| **Race n (%)** |  |  |  |  | 0.8836 | 0.1894 | 0.1421 |
| Caucasian | 2165 (81.45) | 610 (82.99) | 640 (81.12) | 915 (80.69) |  |  |  |
| Black | 67 (2.52) | 18 (2.45) | 21 (2.66) | 28 (2.47) |  |  |  |
| Hispanic | 191 (7.19) | 50 (6.80) | 64 (8.11) | 77 (6.79) |  |  |  |
| Other | 190 (7.15) | 43 (5.85) | 48 (6.08) | 99 (8.73) |  |  |  |
| Unknown | 45 (1.69) | 14 (1.90) | 16 (2.03) | 15 (1.32) |  |  |  |
| **Insurance status n (%)** |  |  |  |  | **<0.0001** | **0.0289** | **<0.0001** |
| Medicare | 461 (17.34) | 131 (17.82) | 95 (12.04) | 235 (20.72) |  |  |  |
| Medicaid | 317 (11.93) | 73 (9.93) | 87 (11.03) | 157 (13.84) |  |  |  |
| Commercial | 1751 (65.88) | 510 (69.39) | 532 (67.43) | 709 (62.52) |  |  |  |
| Uninsured | 125 (4.70) | 20 (2.72) | 73 (9.25) | 32 (2.82) |  |  |  |
| Other | 4 (0.15) | 1 (0.14) | 2 (0.25) | 1 (0.09) |  |  |  |
| Unknown | 0 (0.00) | 0 (0.00) | 0 (0.00) | 0 (0.00) |  |  |  |
| **Comorbidities n (%)** |  |  |  |  |  |  |  |
| Cerebrospinal fluid leak | 13 (0.49) | 4 (0.54) | 3 (0.38) | 6 (0.53) | 0.6361 | 0.9651 | 0.638 |
| Cochlear implant | 13 (0.49) | 5 (0.68) | 4 (0.51) | 4 (0.35) | 0.6591 | 0.3177 | 0.6052 |
| Sickle cell disease/other hemaglobinopathy | 32 (1.20) | 5 (0.68) | 11 (1.39) | 16 (1.41) | 0.1718 | 0.1432 | 0.9755 |
| Congenital or acquired asplenia | 82 (3.09) | 24 (3.27) | 17 (2.15) | 41 (3.62) | 0.1806 | 0.6865 | 0.0654 |
| HIV | 80 (3.01) | 22 (2.99) | 27 (3.42) | 31 (2.73) | 0.6353 | 0.7413 | 0.3853 |
| Chronic renal failure | 338 (12.72) | 72 (9.80) | 79 (10.01) | 187 (16.49) | 0.8875 | **<0.0001** | **<0.0001** |
| Nephrotic syndrome | 74 (2.78) | 25 (3.40) | 17 (2.15) | 32 (2.82) | 0.1374 | 0.4767 | 0.3611 |
| Leukemia | 52 (1.96) | 17 (2.31) | 18 (2.28) | 17 (1.50) | 0.9672 | 0.1985 | 0.2069 |
| Hodgkin disease | 27 (1.02) | 7 (0.95) | 9 (1.14) | 11 (0.97) | 0.7186 | 0.9696 | 0.7167 |
| Lymphoma | 90 (3.39) | 27 (3.67) | 26 (3.30) | 37 (3.26) | 0.6872 | 0.6334 | 0.9686 |
| Generalized malignancy | 1645 (61.89) | 464 (63.13) | 518 (65.65) | 663 (58.47) | 0.3038 | **0.0441** | **0.0014** |
| Iatrogenic immunosuppression | 404 (15.20) | 94 (12.79) | 103 (13.05) | 207 (18.25) | 0.8774 | **0.0017** | **0.0023** |
| Solid organ transplant | 313 (11.78) | 75 (10.20) | 88 (11.15) | 150 (13.23) | 0.5491 | **0.0498** | 0.1743 |
| Multiple myeloma | 25 (0.94) | 2 (0.27) | 6 (0.76) | 17 (1.50) | 0.1874 | **0.0098** | 0.1427 |
| Other | 19 (0.71) | 4 (0.54) | 5 (0.63) | 10 (0.88) | 0.8198 | 0.4083 | 0.5429 |
| **Median Charlson Comorbidity Index** | 3 | 3 | 2 | 3 |  |  |  |
| Mean Charlson Comorbidity Index (SD) | 3.55 (2.95) | 3.35 (2.84) | 3.05 (2.70) | 4.03 (3.12) | **0.0444** | **<0.0001** | **<0.0001** |
| *Comparisons between study clinics (e.g. A vs B) were made with Chi-square tests or ANOVA, as appropriate. | | | | | | | |

| **Table S6. Demographic Characteristics for High-Risk Adults Age 19-64 Years Overall and Stratified by Intervention Group – May 2015 – Apr 2016** | | | | | | | |
| --- | --- | --- | --- | --- | --- | --- | --- |
| **Demographic Characteristics for High-Risk Adults 19-64 Years (Interim Period)** | **Total N=3,449** | **Family Med Clinics (A) N=1,003** | **Family Med Clinics (B) N=1,036** | **Internal Med Clinics (C) N=1,410** | **p-value AvB*** | **p-value AvC*** | **p-value BvC*** |
| **Median age (years)** | 53 | 52 | 52 | 55 | 0.6964 | **<0.0001** | **<0.0001** |
| Mean age (SD) | 50.15 (11.08) | 48.83 (11.60) | 49.02 (11.02) | 51.92 (10.51) |  |  |  |
| **Gender n (%)** |  |  |  |  | 0.5101 | 0.3783 | 0.8603 |
| Male | 1411 (40.91) | 422 (42.07) | 421 (40.64) | 568 (40.28) |  |  |  |
| Female | 2038 (59.09) | 581 (57.93) | 615 (59.36) | 842 (59.72) |  |  |  |
| **Race n (%)** |  |  |  |  | **0.0143** | 0.2754 | **<0.0001** |
| Caucasian | 2755 (79.88) | 813 (81.06) | 814 (78.57) | 1128 (80.00) |  |  |  |
| Black | 83 (2.41) | 28 (2.79) | 29 (2.80) | 26 (1.84) |  |  |  |
| Hispanic | 310 (8.99) | 78 (7.78) | 119 (11.49) | 113 (8.01) |  |  |  |
| Other | 247 (7.16) | 70 (6.98) | 52 (5.02) | 125 (8.87) |  |  |  |
| Unknown | 54 (1.57) | 14 (1.40) | 22 (2.12) | 18 (1.28) |  |  |  |
| Insurance status n (%) |  |  |  |  | **<0.0001** | 0.0865 | **<0.0001** |
| Medicare | 647 (18.76) | 190 (18.94) | 156 (15.06) | 301 (21.35) |  |  |  |
| Medicaid | 395 (11.45) | 98 (9.77) | 131 (12.64) | 166 (11.77) |  |  |  |
| Commercial | 2252 (65.29) | 696 (69.39) | 636 (61.39) | 920 (65.25) |  |  |  |
| Uninsured | 152 (4.41) | 17 (1.69) | 112 (10.81) | 23 (1.63) |  |  |  |
| Other | 3 (0.09) | 2 (0.20) | 1 (0.10) | 0 (0.00) |  |  |  |
| Unknown | 0 (0.00) | 0 (0.00) | 0 (0.00) | 0 (0.00) |  |  |  |
| **Comorbidities n (%)** |  |  |  |  |  |  |  |
| Cerebrospinal fluid leak | 9 (0.26) | 1 (0.10) | 1 (0.10) | 7 (0.50) | 0.9817 | 0.0947 | 0.0869 |
| Cochlear implant | 19 (0.55) | 7 (0.70) | 6 (0.58) | 6 (0.43) | 0.7363 | 0.3677 | 0.5911 |
| Sickle cell disease/other hemaglobinopathy | 34 (0.99) | 8 (0.80) | 12 (1.16) | 14 (0.99) | 0.4087 | 0.6189 | 0.6935 |
| Congenital or acquired asplenia | 110 (3.19) | 27 (2.69) | 28 (2.70) | 55 (3.90) | 0.988 | 0.1063 | 0.1059 |
| HIV | 86 (2.49) | 27 (2.69) | 30 (2.90) | 29 (2.06) | 0.7801 | 0.3071 | 0.1814 |
| Chronic renal failure | 427 (12.38) | 90 (8.97) | 115 (11.10) | 222 (15.74) | 0.1103 | **<0.0001** | **0.001** |
| Nephrotic syndrome | 85 (2.46) | 27 (2.69) | 18 (1.74) | 40 (2.84) | 0.1425 | 0.8309 | 0.0774 |
| Leukemia | 78 (2.26) | 17 (1.69) | 28 (2.70) | 33 (2.34) | 0.1215 | 0.2726 | 0.5702 |
| Hodgkin disease | 32 (0.93) | 9 (0.90) | 9 (0.87) | 14 (0.99) | 0.945 | 0.8117 | 0.7532 |
| Lymphoma | 127 (3.68) | 39 (3.89) | 34 (3.28) | 54 (3.83) | 0.4612 | 0.9413 | 0.4721 |
| Generalized malignancy | 1842 (53.41) | 541 (53.94) | 581 (56.08) | 720 (51.06) | 0.3308 | 0.1636 | **0.014** |
| Iatrogenic immunosuppression | 1003 (29.08) | 287 (28.61) | 292 (28.19) | 424 (30.07) | 0.83 | 0.4392 | 0.3112 |
| Solid organ transplant | 412 (11.95) | 110 (10.97) | 110 (10.62) | 192 (13.62) | 0.7993 | 0.0525 | **0.0259** |
| Multiple myeloma | 27 (0.78) | 3 (0.30) | 12 (1.16) | 12 (0.85) | **0.0232** | 0.0891 | 0.4462 |
| Other | 23 (0.67) | 5 (0.50) | 5 (0.48) | 13 (0.92) | 0.9591 | 0.2335 | 0.209 |
| **Median Charlson Comorbidity Index** | 3 | 3 | 3 | 3 |  |  |  |
| Mean Charlson Comorbidity Index (SD) | 3.78 (2.96) | 3.54 (2.87) | 3.43 (2.78) | 4.20 (3.10) | 0.3955 | **<0.0001** | **<0.0001** |
| *Comparisons between study clinics (e.g. A vs B) were made with Chi-square tests or ANOVA, as appropriate. | | | | | | | |

| **Table S7. Demographic Characteristics for High-Risk Adults Age 19-64 Years Overall and Stratified by Intervention Group – May 2016 – Jul 2017** | | | | | | | |  |
| --- | --- | --- | --- | --- | --- | --- | --- | --- |
| **Demographic Characteristics for High-Risk Adults 19-64 Years (Follow-up Period)** | **Total N=3,612** | **Family Med Clinics (A) N=1,057** | **Family Med Clinics (B) N=1,151** | **Internal Med Clinics (C) N=1,404** | **p-value AvB*** | **p-value AvC*** | **p-value BvC*** |  |
| **Median age (years)** | 53 | 51 | 50 | 55 | 0.9591 | **<0.0001** | **<0.0001** |  |
| Mean age (SD) | 49.75 (11.26) | 48.37 (11.75) | 48.40 (11.34) | 51.91 (10.46) |  |  |  |  |
| **Gender n (%)** |  |  |  |  | 0.5744 | 0.5257 | 0.2109 |  |
| Male | 1402 (38.82) | 409 (38.69) | 432 (37.53) | 561 (39.96) |  |  |  |  |
| Female | 2210 (61.18) | 648 (61.31) | 719 (62.47) | 843 (60.04) |  |  |  |  |
| **Race n (%)** |  |  |  |  | **0.0175** | **0.0109** | **0.0003** |  |
| Caucasian | 2808 (77.74) | 850 (80.42) | 883 (76.72) | 1075 (76.57) |  |  |  |  |
| Black | 93 (2.57) | 30 (2.84) | 30 (2.61) | 33 (2.35) |  |  |  |  |
| Hispanic | 358 (9.91) | 94 (8.89) | 140 (12.16) | 124 (8.83) |  |  |  |  |
| Other | 309 (8.55) | 76 (7.19) | 78 (6.78) | 155 (11.04) |  |  |  |  |
| Unknown | 44 (1.22) | 7 (0.66) | 20 (1.74) | 17 (1.21) |  |  |  |  |
| **Insurance status n (%)** |  |  |  |  | **<0.0001** | 0.0528 | **<0.0001** |  |
| Medicare | 684 (18.94) | 195 (18.45) | 191 (16.59) | 298 (21.23) |  |  |  |  |
| Medicaid | 385 (10.66) | 99 (9.37) | 126 (10.95) | 160 (11.40) |  |  |  |  |
| Commercial | 2386 (66.06) | 742 (70.20) | 732 (63.60) | 912 (64.96) |  |  |  |  |
| Uninsured | 149 (4.13) | 21 (1.99) | 99 (8.60) | 29 (2.07) |  |  |  |  |
| Other | 2 (0.06) | 0 (0.00) | 1 (0.09) | 1 (0.07) |  |  |  |  |
| Unknown | 6 (0.17) | 0 (0.00) | 2 (0.17) | 2 (0.17) |  |  |  |  |
| **Comorbidities n (%)** |  |  |  |  |  |  |  | |
| Cerebrospinal fluid leak | 18 (0.50) | 4 (0.38) | 5 (0.43) | 9 (0.64) | 0.8366 | 0.3737 | 0.4815 | |
| Cochlear implant | 24 (0.66) | 4 (0.38) | 12 (1.04) | 8 (0.57) | 0.0661 | 0.4999 | 0.1773 | |
| Sickle cell disease/other hemaglobinopathy | 54 (1.50) | 12 (1.14) | 22 (1.91) | 20 (1.42) | 0.139 | 0.5307 | 0.3356 | |
| Congenital or acquired asplenia | 120 (3.32) | 26 (2.46) | 31 (2.69) | 63 (4.49) | 0.7296 | **0.0077** | **0.0165** | |
| HIV | 98 (2.71) | 24 (2.27) | 41 (3.56) | 33 (2.35) | 0.0729 | 0.8963 | 0.0692 | |
| Chronic renal failure | 482 (13.34) | 114 (10.79) | 126 (10.95) | 242 (17.24) | 0.9029 | **<0.0001** | **<0.0001** | |
| Nephrotic syndrome | 90 (2.49) | 31 (2.93) | 17 (1.48) | 42 (2.99) | **0.0191** | 0.9324 | **0.0112** | |
| Leukemia | 95 (2.63) | 30 (2.84) | 34 (2.95) | 31 (2.21) | 0.8714 | 0.3195 | 0.2335 | |
| Hodgkin disease | 32 (0.89) | 12 (1.14) | 10 (0.87) | 10 (0.71) | 0.5288 | 0.2697 | 0.655 | |
| Lymphoma | 120 (3.32) | 34 (3.22) | 41 (3.56) | 45 (3.21) | 0.6544 | 0.9872 | 0.6186 | |
| Generalized malignancy | 1741 (48.20) | 529 (50.05) | 559 (48.57) | 653 (46.51) | 0.4869 | 0.0821 | 0.3003 | |
| Iatrogenic immunosuppression | 1089 (30.15) | 308 (29.14) | 334 (29.02) | 447 (31.84) | 0.9502 | 0.1507 | 0.1238 | |
| Solid organ transplant | 451 (12.49) | 125 (11.83) | 135 (11.73) | 191 (13.60) | 0.9437 | 0.1918 | 0.1575 | |
| Multiple myeloma | 38 (1.05) | 10 (0.95) | 13 (1.13) | 15 (1.07) | 0.6716 | 0.7646 | 0.8827 | |
| Other | 30 (0.83) | 11 (1.04) | 6 (0.52) | 13 (0.93) | 0.1631 | 0.7743 | 0.2362 | |
| **Median Charlson Comorbidity Index** | 3 | 3 | 3 | 4 |  |  |  | |
| Mean Charlson Comorbidity Index (SD) | 4.01 (3.00) | 3.78 (2.81) | 3.68 (2.92) | 4.45 (3.16) | 0.4461 | **<0.0001** | **<0.0001** | |
| *Comparisons between study clinics (e.g. A vs B) were made with Chi-square tests or ANOVA, as appropriate. | | | | | | | | |

| **Table S8. Demographic Characteristics for Immunocompetent Adults Aged 65+ Overall and Stratified by Intervention Group - Aug 2013 – Jul 2014** | | | | | | | |
| --- | --- | --- | --- | --- | --- | --- | --- |
| **Demographic Characteristics for Immunocompetent Adults aged 65+ (Baseline Period)** | **Total N=9,480** | **Family Med Clinics (A) N=2,162** | **Family Med Clinics (B) N=2,519** | **Internal Med Clinics (C) N=4,799** | **p-value AvB*** | **p-value AvC*** | **p-value BvC*** |
| **Median age (years)** | 72 | 71 | 71 | 73 |  |  |  |
| Mean age (SD) | 73.49 (7.19) | 73.21 (7.21) | 72.55 (6.77) | 74.12 (7.33) | **0.0015** | **<0.0001** | **<0.0001** |
| **Gender n (%)** |  |  |  |  | **0.0003** | 0.6399 | **<0.0001** |
| Male | 3698 (39.01) | 820 (37.93) | 1086 (43.11) | 1792 (37.34) |  |  |  |
| Female | 5782 (60.99) | 1342 (62.07) | 1433 (56.89) | 3007 (62.66) |  |  |  |
| **Race n (%)** |  |  |  |  | **<0.0001** | 0.4477 | **<0.0001** |
| Caucasian | 7517 (79.29) | 1703 (78.77) | 1961 (77.85) | 3853 (80.29) |  |  |  |
| Black | 109 (1.15) | 24 (1.11) | 27 (1.07) | 58 (1.21) |  |  |  |
| Hispanic | 888 (9.37) | 185 (8.56) | 318 (12.62) | 385 (8.02) |  |  |  |
| Other | 780 (8.23) | 202 (9.34) | 157 (6.23) | 421 (8.77) |  |  |  |
| Unknown | 186 (1.96) | 48 (2.22) | 56 (2.22) | 82 (1.71) |  |  |  |
| **Insurance status n (%)** |  |  |  |  | **<0.0001** | **0.0158** | **<0.0001** |
| Medicare | 7780 (82.07) | 1748 (80.85) | 2002 (79.48) | 4030 (83.98) |  |  |  |
| Medicaid | 160 (1.69) | 45 (2.08) | 44 (1.75) | 71 (1.48) |  |  |  |
| Commercial | 1370 (14.45) | 350 (16.19) | 367 (14.57) | 653 (13.61) |  |  |  |
| Uninsured | 153 (1.61) | 15 (0.69) | 100 (3.97) | 38 (0.79) |  |  |  |
| Other | 17 (0.18) | 4 (0.19) | 6 (0.24) | 7 (0.15) |  |  |  |
| Unknown | 0 (0.00) | 0 (0.00) | 0 (0.00) | 0 (0.00) |  |  |  |
| **Comorbidities n (%)** |  |  |  |  |  |  |  |
| Chronic heart disease | 2028 (21.39) | 464 (21.46) | 378 (15.01) | 1186 (24.71) | **<0.0001** | **0.0032** | **<0.0001** |
| Chronic lung disease | 1857 (19.59) | 421 (19.47) | 367 (14.57) | 1069 (22.28) | **<0.0001** | **0.0083** | **<0.0001** |
| Diabetes mellitus | 2343 (24.72) | 524 (24.24) | 565 (22.43) | 1254 (26.13) | 0.1446 | 0.0937 | **0.0005** |
| Cerebrospinal fluid leak | 0 | 0 | 0 | 0 |  |  |  |
| Cochlear implant | 0 | 0 | 0 | 0 |  |  |  |
| Alcoholism | 8 (0.08) | 2 (0.09) | 1 (0.04) | 5 (0.10) | 0.4766 | 0.8869 | 0.3598 |
| Chronic liver disease, cirrhosis | 427 (4.50) | 91 (4.21) | 92 (3.65) | 244 (5.08) | 0.3271 | 0.1144 | **0.0054** |
| Cigarette smoking | 574 (6.05) | 159 (7.35) | 139 (5.52) | 276 (5.75) | **0.0103** | **0.0106** | 0.682 |
| Other | 10 (0.11) | 3 (0.14) | 0 (0.00) | 7 (0.15) | 0.0615 | 0.9423 | 0.0551 |
| **Median Charlson Comorbidity Index** | 1 | 1 | 1 | 2 |  |  |  |
| Mean Charlson Comorbidity Index (SD) | 1.90 (2.15) | 1.85 (2.06) | 1.44 (1.87) | 2.16 (2.28) | **<0.0001** | **<0.0001** | **<0.0001** |
| *Comparisons between study clinics (e.g. A vs B) were made with Chi-square tests or ANOVA, as appropriate. | | | | | | | |

| **Table S9. Demographic Characteristics for Immunocompetent Adults Aged 65+ Overall and Stratified by Intervention Group - May 2015 - Apr 2016** | | | | | | | |
| --- | --- | --- | --- | --- | --- | --- | --- |
| **Demographic Characteristics for Immunocompetent Adults Aged 65+ (Interim Period)** | **Total N=11,318** | **Family Med Clinics (A) N=2,597** | **Family Med Clinics (B) N=3,119** | **Internal Med Clinics (C) N=5,602** | **p-value AvB*** | **p-value AvC*** | **p-value BvC*** |
| **Median age (years)** | 71 | 71 | 71 | 72 |  |  |  |
| Mean age (SD) | 73.19 (7.06) | 73.02 (7.09) | 72.38 (6.71) | 73.73 (7.19) | **0.0005** | **<0.0001** | **<0.0001** |
| **Gender n (%)** |  |  |  |  | **0.0002** | 0.5085 | **0.0001** |
| Male | 4555 (40.25) | 1000 (38.51) | 1355 (43.44) | 2200 (39.27) |  |  |  |
| Female | 6763 (59.75) | 1597 (61.49) | 1764 (56.56) | 3402 (60.73) |  |  |  |
| **Race n (%)** |  |  |  |  | **<0.0001** | 0.5373 | **<0.0001** |
| Caucasian | 8997 (79.49) | 2077 (79.98) | 2414 (77.40) | 4506 (80.44) |  |  |  |
| Black | 125 (1.10) | 30 (1.16) | 31 (0.99) | 64 (1.14) |  |  |  |
| Hispanic | 1060 (9.37) | 193 (7.43) | 422 (13.53) | 445 (7.94) |  |  |  |
| Other | 932 (8.23) | 253 (9.74) | 194 (6.22) | 485 (8.66) |  |  |  |
| Unknown | 204 (1.80) | 44 (1.69) | 58 (1.86) | 102 (1.82) |  |  |  |
| **Insurance status n (%)** |  |  |  |  | **<0.0001** | **0.023** | **<0.0001** |
| Medicare | 9342 (82.54) | 2124 (81.79) | 2502 (80.22) | 4716 (84.18) |  |  |  |
| Medicaid | 124 (1.10) | 20 (0.77) | 47 (1.51) | 57 (1.02) |  |  |  |
| Commercial | 1657 (14.64) | 434 (16.71) | 439 (14.08) | 784 (14.00) |  |  |  |
| Uninsured | 178 (1.57) | 18 (0.69) | 123 (3.94) | 37 (0.66) |  |  |  |
| Other | 16 (0.14) | 1 (0.04) | 8 (0.26) | 7 (0.12) |  |  |  |
| Unknown | 1 (0.01) | 0 (0.00) | 0 (0.00) | 0 (0.00) |  |  |  |
| **Comorbidities n (%)** |  |  |  |  |  |  |  |
| Chronic heart disease | 2460 (21.74) | 559 (21.52) | 533 (17.09) | 1368 (24.42) | **<0.0001** | **0.004** | **<0.0001** |
| Chronic lung disease | 2207 (19.50) | 485 (18.68) | 509 (16.32) | 1213 (21.65) | **0.0193** | **0.002** | **<0.0001** |
| Diabetes mellitus | 2777 (24.54) | 620 (23.87) | 722 (23.15) | 1435 (25.62) | 0.5195 | 0.0904 | **0.0105** |
| Cerebrospinal fluid leak | 0 | 0 | 0 | 0 |  |  |  |
| Cochlear implant | 0 | 0 | 0 | 0 |  |  |  |
| Alcoholism | 12 (0.11) | 2 (0.08) | 3 (0.10) | 7 (0.12) | 0.8071 | 0.5419 | 0.7036 |
| Chronic liver disease, cirrhosis | 545 (4.82) | 104 (4.00) | 127 (4.07) | 314 (5.61) | 0.8978 | **0.0022** | **0.0017** |
| Cigarette smoking | 640 (5.65) | 170 (6.55) | 170 (5.45) | 300 (5.36) | 0.0812 | **0.0309** | 0.8503 |
| Other | 9 (0.08) | 3 (0.12) | 1 (0.03) | 5 (0.09) | 0.2348 | 0.7231 | 0.3289 |
| **Median Charlson Comorbidity Index** | 1 | 1 | 1 | 2 |  |  |  |
| Mean Charlson Comorbidity Index (SD) | 1.97 (2.22) | 1.93 (2.15) | 1.63 (2.00) | 2.18 (2.34) | **<0.0001** | **<0.0001** | **<0.0001** |
| *Comparisons between study clinics (e.g. A vs B) were made with Chi-square tests or ANOVA, as appropriate. | | | | | | | |

| **Table S10. Demographic Characteristics for Immunocompetent Adults Aged 65+ Overall and Stratified by Intervention Group - May 2016 – July 2017** | | | | | | | | |
| --- | --- | --- | --- | --- | --- | --- | --- | --- |
| **Demographic Characteristics for Immunocompetent Adults Aged 65+ (Follow-up Period)** | **Total N=11,318** | **Family Med Clinics (A) N=2,597** | **Family Med Clinics (B) N=3,119** | **Internal Med Clinics (C) N=5,602** | **p-value AvB*** | **p-value AvC*** | | **p-value BvC*** |
| **Median age (years)** | 71 | 71 | 71 | 72 |  |  | |  |
| Mean age (SD) | 73.13 (7.02) | 72.84 (7.04) | 72.50 (6.68) | 73.70 (7.18) | **0.0438** | **<0.0001** | | **<0.0001** |
| **Gender n (%)** |  |  |  |  | **<0.0001** | 0.2477 | | **0.0002** |
| Male | 5353 (40.12) | 1187 (38.03) | 1752 (43.01) | 2414 (39.27) |  |  | |  |
| Female | 7988 (59.88) | 1934 (61.97) | 2321 (56.99) | 3733 (60.73) |  |  | |  |
| **Race n (%)** |  |  |  |  | **<0.0001** | 0.731 | | **<0.0001** |
| Caucasian | 10697 (80.18) | 2515 (80.58) | 3197 (78.49) | 4985 (81.10) |  |  | |  |
| Black | 141 (1.06) | 29 (0.93) | 42 (1.03) | 70 (1.14) |  |  | |  |
| Hispanic | 1227 (9.20) | 250 (8.01) | 517 (12.69) | 460 (7.48) |  |  | |  |
| Other | 1052 (7.89) | 279 (8.94) | 242 (5.94) | 531 (8.64) |  |  | |  |
| Unknown | 224 (1.68) | 48 (1.54) | 75 (1.84) | 101 (1.64) |  |  | |  |
| **Insurance status n (%)** |  |  |  |  | **<0.0001** | **0.0004** | | **<0.0001** |
| Medicare | 10960 (82.15) | 2538 (81.32) | 3252 (79.84) | 5170 (84.11) |  |  | |  |
| Medicaid | 169 (1.27) | 27 (0.87) | 60 (1.47) | 82 (1.33) |  |  | |  |
| Commercial | 1978 (14.83) | 513 (16.44) | 623 (15.30) | 842 (13.70) |  |  | |  |
| Uninsured | 209 (1.57) | 37 (1.19) | 126 (3.09) | 46 (0.75) |  |  | |  |
| Other | 17 (0.13) | 3 (0.10) | 9 (0.22) | 5 (0.08) |  |  | |  |
| Unknown | 8 (0.06) | 3 (0.10) | 3 (0.07) | 3 (0.07) |  |  | |  |
| **Comorbidities n (%)** |  |  |  |  |  |  | |  |
| Chronic heart disease | 2798 (20.97) | 635 (20.35) | 674 (16.55) | 1489 (24.22) | **<0.0001** | **<0.0001** | | **<0.0001** |
| Chronic lung disease | 2718 (20.37) | 636 (20.38) | 668 (16.40) | 1414 (23.00) | **<0.0001** | **0.004** | | **<0.0001** |
| Diabetes mellitus | 3334 (24.99) | 775 (24.83) | 972 (23.86) | 1587 (25.82) | 0.343 | 0.3034 | | **0.0257** |
| Cerebrospinal fluid leak | 0 | 0 | 0 | 0 |  |  | |  |
| Cochlear implant | 0 | 0 | 0 | 0 |  |  | |  |
| Alcoholism | 14 (0.10) | 3 (0.10) | 1 (0.02) | 10 (0.16) | 0.2019 | 0.4184 | | **0.0371** |
| Chronic liver disease, cirrhosis | 686 (5.14) | 136 (4.36) | 188 (4.62) | 362 (5.89) | 0.6008 | **0.002** | | **0.0052** |
| Cigarette smoking | 766 (5.74) | 242 (7.75) | 193 (4.74) | 331 (5.38) | **<0.0001** | **<0.0001** | | 0.147 |
| Other | 9 (0.07) | 3 (0.10) | 2 (0.05) | 4 (0.07) | 0.4533 | 0.6071 | | 0.7442 |
| **Median Charlson Comorbidity Index** | 1 | 1 | 1 | 2 |  |  | |  |
| Mean Charlson Comorbidity Index (SD) | 2.00 (2.24) | 1.96 (2.17) | 1.66 (2.06) | 2.24 (2.37) | **<0.0001** | **<0.0001** | | **<0.0001** |
| *Comparisons between study clinics (e.g. A vs B) were made with Chi-square tests or ANOVA, as appropriate. | | | | | | |  |  |

| **Table S11. Demographic Characteristics for Immunocompromised Adults Aged 65+ Overall and Stratified by Intervention Group - Aug 2013 – Jul 2014** | | | | | | | |
| --- | --- | --- | --- | --- | --- | --- | --- |
| **Demographic Characteristics for Immunocompromised Adults Aged 65+ (Baseline Period)** | **Total N=2,577** | **Family Med Clinics (A) N=516** | **Family Med Clinics (B) N=512** | **Internal Med Clinics (C) N=1,549** | **p-value AvB*** | **p-value AvC*** | **p-value BvC*** |
| **Median age (years)** | 74 | 73.5 | 73 | 74 |  |  |  |
| Mean age (SD) | 75.11 (7.61) | 74.71 (7.69) | 74.26 (7.41) | 75.53 (7.63) | 0.3493 | **0.0328** | **0.0011** |
| **Gender n (%)** |  |  |  |  | 0.0795 | 0.8687 | **0.0478** |
| Male | 1308 (50.76) | 255 (49.42) | 281 (54.88) | 772 (49.84) |  |  |  |
| Female | 1269 (49.24) | 261 (50.58) | 231 (45.12) | 777 (50.16) |  |  |  |
| **Race n (%)** |  |  |  |  | **0.0449** | 0.8484 | **0.0021** |
| Caucasian | 2226 (86.38) | 444 (86.05) | 430 (83.98) | 1352 (87.28) |  |  |  |
| Black | 17 (0.66) | 5 (0.97) | 1 (0.20) | 11 (0.71) |  |  |  |
| Hispanic | 165 (6.40) | 30 (5.81) | 52 (10.16) | 83 (5.36) |  |  |  |
| Other | 140 (5.43) | 32 (6.20) | 25 (4.88) | 83 (5.36) |  |  |  |
| Unknown | 29 (1.13) | 5 (0.97) | 4 (0.78) | 20 (1.29) |  |  |  |
| **Insurance Status n (%)** |  |  |  |  | **0.0116** | 0.8056 | **<0.0001** |
| Medicare | 2213 (85.88) | 441 (85.47) | 434 (84.77) | 1338 (86.38) |  |  |  |
| Medicaid | 16 (0.62) | 3 (0.58) | 4 (0.78) | 9 (0.58) |  |  |  |
| Commercial | 331 (12.84) | 71 (13.76) | 61 (11.91) | 199 (12.85) |  |  |  |
| Uninsured | 14 (0.54) | 0 (0.00) | 12 (2.34) | 2 (0.13) |  |  |  |
| Other | 3 (0.12) | 1 (0.19) | 1 (0.20) | 1 (0.06) |  |  |  |
| Unknown | 0 (0.00) | 0 (0.00) | 0 (0.00) | 0 (0.00) |  |  |  |
| **Comorbidities n (%)** |  |  |  |  |  |  |  |
| Cerebrospinal fluid leak | 1 (0.04) | 0 (0.00) | 0 (0.00) | 1 (0.06) |  | 0.5637 | 0.5652 |
| Cochlear implant | 8 (0.31) | 3 (0.58) | 1 (0.20) | 4 (0.26) | 0.3201 | 0.274 | 0.8019 |
| Sickle cell disease/other hemaglobinopathy | 6 (0.23) | 0 (0.00) | 1 (0.20) | 5 (0.32) | 0.3152 | 0.1963 | 0.6425 |
| Congenital or acquired asplenia | 35 (1.36) | 4 (0.78) | 6 (1.17) | 25 (1.61) | 0.517 | 0.1608 | 0.4762 |
| HIV | 6 (0.23) | 0 (0.00) | 3 (0.59) | 3 (0.19) | 0.0816 | 0.3171 | 0.1532 |
| Chronic renal failure | 375 (14.55) | 75 (14.53) | 67 (13.09) | 233 (15.04) | 0.5008 | 0.7794 | 0.2766 |
| Nephrotic syndrome | 24 (0.93) | 5 (0.97) | 3 (0.59) | 16 (1.03) | 0.4847 | 0.9002 | 0.3589 |
| Leukemia | 63 (2.44) | 7 (1.36) | 13 (2.54) | 43 (2.78) | 0.1699 | 0.0693 | 0.775 |
| Hodgkin disease | 5 (0.19) | 0 (0.00) | 1 (0.20) | 4 (0.26) | 0.3152 | 0.2479 | 0.8019 |
| Lymphoma | 94 (3.65) | 19 (3.68) | 21 (4.10) | 54 (3.49) | 0.7281 | 0.8346 | 0.5191 |
| Generalized malignancy | 1893 (73.46) | 388 (75.19) | 390 (76.17) | 1115 (71.98) | 0.7147 | 0.1557 | 0.064 |
| Iatrogenic immunosuppression | 229 (8.89) | 36 (6.98) | 37 (7.23) | 156 (10.07) | 0.8761 | **0.0361** | 0.0555 |
| Solid organ transplant | 248 (9.62) | 43 (8.33) | 49 (9.57) | 156 (10.07) | 0.4873 | 0.2467 | 0.7428 |
| Multiple myeloma | 42 (1.63) | 4 (0.78) | 7 (1.37) | 31 (2.00) | 0.3563 | 0.0617 | 0.3552 |
| Other | 9 (0.35) | 2 (0.39) | 2 (0.39) | 5 (0.32) | 0.9938 | 0.8264 | 0.8191 |
| **Median Charlson Comorbidity Index** | 4 | 4 | 3 | 5 |  |  |  |
| Mean Charlson Comorbidity Index (SD) | 4.77 (3.20) | 4.62 (3.23) | 4.22 (3.25) | 5.01 (3.15) | **0.0426** | **0.0181** | **<0.0001** |
| *Comparisons between study clinics (e.g. A vs B) were made with Chi-square tests or ANOVA, as appropriate. | | | | | | | |

| **Table S12. Demographic Characteristics for Immunocompromised Adults Aged 65+ Overall and Stratified by Intervention Group - May 2015 - Apr 2016** | | | | | | | | |
| --- | --- | --- | --- | --- | --- | --- | --- | --- |
| **Demographic Characteristics for Immunocompromised Adults Aged 65+ (Interim Period)** | **Total N=3,200** | **Family Med Clinics (A) N=608** | **Family Med Clinics (B) N=654** | **Internal Med Clinics (C) N=1,938** | | **p-value AvB*** | **p-value AvC*** | **p-value BvC*** |
| **Median age (years)** | 74 | 73 | 72 | 74 | |  |  |  |
| Mean age (SD) | 74.81 (7.30) | 74.37 (7.24) | 73.49 (6.92) | 75.39 (7.38) | | **0.0323** | **0.0025** | **<0.0001** |
| **Gender n (%)** |  |  |  |  | | **0.0144** | 0.5844 | **0.0129** |
| Male | 1596 (49.88) | 290 (47.70) | 357 (54.59) | 949 (48.97) | |  |  |  |
| Female | 1604 (50.13) | 318 (52.30) | 297 (45.41) | 989 (51.03) | |  |  |  |
| **Race n (%)** |  |  |  |  | | **0.0075** | 0.1714 | **0.0027** |
| Caucasian | 2767 (86.47) | 520 (85.53) | 552 (84.40) | 1695 (87.46) | |  |  |  |
| Black | 20 (0.63) | 9 (1.48) | 1 (0.15) | 10 (0.52) | |  |  |  |
| Hispanic | 195 (6.09) | 34 (5.59) | 61 (9.33) | 100 (5.16) | |  |  |  |
| Other | 180 (5.63) | 38 (6.25) | 32 (4.89) | 110 (5.68) | |  |  |  |
| Unknown | 38 (1.19) | 7 (1.15) | 8 (1.22) | 23 (1.19) | |  |  |  |
| **Insurance status n (%)** |  |  |  |  | | **0.0236** | 0.5389 | **0.0005** |
| Medicare | 2774 (86.69) | 533 (87.66) | 562 (85.93) | 1679 (86.64) | |  |  |  |
| Medicaid | 9 (0.28) | 0 (0.00) | 3 (0.46) | 6 (0.31) | |  |  |  |
| Commercial | 393 (12.28) | 73 (12.01) | 75 (11.47) | 245 (12.64) | |  |  |  |
| Uninsured | 19 (0.59) | 2 (0.33) | 12 (1.83) | 5 (0.26) | |  |  |  |
| Other | 5 (0.16) | 0 (0.00) | 2 (0.31) | 3 (0.15) | |  |  |  |
| Unknown | 0 (0.00) | 0 (0.00) | 0 (0.00) | 0 (0.00) | |  |  |  |
| **Comorbidities n (%)** |  |  |  | |  |  |  |  |
| Cerebrospinal fluid leak | 2 (0.06) | 0 (0.00) | 1 (0.15) | | 1 (0.05) | 0.3348 | 0.5753 | 0.4198 |
| Cochlear implant | 14 (0.44) | 3 (0.49) | 2 (0.31) | | 9 (0.46) | 0.596 | 0.9274 | 0.5896 |
| Sickle cell disease/other hemaglobinopathy | 5 (0.16) | 0 (0.00) | 1 (0.15) | | 4 (0.21) | 0.3348 | 0.2622 | 0.7875 |
| Congenital or acquired asplenia | 39 (1.22) | 6 (0.99) | 5 (0.76) | | 28 (1.44) | 0.6712 | 0.3907 | 0.1797 |
| HIV | 7 (0.22) | 2 (0.33) | 1 (0.15) | | 4 (0.21) | 0.5211 | 0.5866 | 0.7875 |
| Chronic renal failure | 433 (13.53) | 78 (12.83) | 80 (12.23) | | 275 (14.19) | 0.749 | 0.3969 | 0.208 |
| Nephrotic syndrome | 33 (1.03) | 6 (0.99) | 5 (0.76) | | 22 (1.14) | 0.6712 | 0.7596 | 0.4195 |
| Leukemia | 94 (2.94) | 12 (1.97) | 21 (3.21) | | 61 (3.15) | 0.1687 | 0.1302 | 0.9361 |
| Hodgkin disease | 6 (0.19) | 0 (0.00) | 1 (0.15) | | 5 (0.26) | 0.3348 | 0.21 | 0.6287 |
| Lymphoma | 109 (3.41) | 23 (3.78) | 23 (3.52) | | 63 (3.25) | 0.801 | 0.5263 | 0.7426 |
| Generalized malignancy | 2264 (70.75) | 423 (69.57) | 468 (71.56) | | 1373 (70.85) | 0.4388 | 0.5477 | 0.728 |
| Iatrogenic immunosuppression | 541 (16.91) | 95 (15.63) | 105 (16.06) | | 341 (17.60) | 0.8344 | 0.2605 | 0.3668 |
| Solid organ transplant | 318 (9.94) | 53 (8.72) | 59 (9.02) | | 206 (10.63) | 0.8494 | 0.1735 | 0.2405 |
| Multiple myeloma | 55 (1.72) | 8 (1.32) | 13 (1.99) | | 34 (1.75) | 0.3511 | 0.4588 | 0.6989 |
| Other | 13 (0.41) | 3 (0.49) | 1 (0.15) | | 9 (0.46) | 0.2822 | 0.9274 | 0.2665 |
| **Median Charlson Comorbidity Index** | 4 | 4 | 4 | | 5 |  |  |  |
| Mean Charlson Comorbidity Index (SD) | 5.03 (3.36) | 4.76 (3.39) | 4.59 (3.23) | | 5.27 (3.38) | 0.3558 | **0.0011** | **<0.0001** |
| *Comparisons between study clinics (e.g. A vs B) were made with Chi-square tests or ANOVA, as appropriate. | | | | | | | | |

| **Table S13. Demographic Characteristics for Immunocompromised Adults Aged 65+ Overall and Stratified by Intervention Group - May 2016 - Jul 2017** | | | | | | | |
| --- | --- | --- | --- | --- | --- | --- | --- |
| **Demographic Characteristics for Immunocompromised Adults Aged 65+ (Follow-up Period)** | **Total N=3,348** | **Family Med Clinics (A) N=651** | **Family Med Clinics (B) N=762** | **Internal Med Clinics (C) N=1,935** | **p-value AvB*** | **p-value AvC*** | **p-value BvC*** |
| **Median age (years)** | 73 | 72 | 72 | 74 |  |  |  |
| Mean age (SD) | 74.60 (7.21) | 73.98 (7.19) | 73.77 (6.99) | 75.14 (7.26) | 0.5855 | **0.0004** | **<0.0001** |
| **Gender n (%)** |  |  |  |  | 0.9646 | 0.3747 | 0.3194 |
| Male | 1654 (49.40) | 329 (50.54) | 386 (50.66) | 939 (48.53) |  |  |  |
| Female | 1694 (50.60) | 322 (49.46) | 376 (49.34) | 996 (51.47) |  |  |  |
| **Race n (%)** |  |  |  |  | **0.0002** | **0.0017** | **<0.0001** |
| Caucasian | 2848 (85.07) | 539 (82.80) | 628 (82.41) | 1681 (86.87) |  |  |  |
| Black | 35 (1.05) | 17 (2.61) | 3 (0.39) | 15 (0.78) |  |  |  |
| Hispanic | 226 (6.75) | 44 (6.76) | 86 (11.29) | 96 (4.96) |  |  |  |
| Other | 205 (6.12) | 44 (6.76) | 40 (5.25) | 121 (6.25) |  |  |  |
| Unknown | 34 (1.02) | 7 (1.08) | 5 (0.66) | 22 (1.14) |  |  |  |
| **Insurance status n (%)** |  |  |  |  | 0.3562 | 0.14 | 0.0993 |
| Medicare | 2909 (86.89) | 556 (85.41) | 656 (86.09) | 1697 (87.70) |  |  |  |
| Medicaid | 23 (0.69) | 4 (0.61) | 7 (0.92) | 12 (0.62) |  |  |  |
| Commercial | 390 (11.65) | 83 (12.75) | 89 (11.68) | 218 (11.27) |  |  |  |
| Uninsured | 19 (0.57) | 4 (0.61) | 9 (1.18) | 6 (0.31) |  |  |  |
| Other | 3 (0.09) | 1 (0.15) | 1 (0.13) | 1 (0.05) |  |  |  |
| Unknown | 4 (0.12) | 3 (0.46) | 0 (0.00) | 0 (0.00) |  |  |  |
| **Comorbidities n (%)** |  |  |  |  |  |  |  |
| Cerebrospinal fluid leak | 6 (0.18) | 0 (0.00) | 2 (0.26) | 4 (0.21) | 0.1908 | 0.2457 | 0.782 |
| Cochlear implant | 20 (0.60) | 4 (0.61) | 5 (0.66) | 11 (0.57) | 0.9217 | 0.8937 | 0.7895 |
| Sickle cell disease/other hemaglobinopathy | 9 (0.27) | 0 (0.00) | 0 (0.00) | 9 (0.47) |  | 0.0813 | 0.0593 |
| Congenital or acquired asplenia | 47 (1.40) | 8 (1.23) | 10 (1.31) | 29 (1.50) | 0.8891 | 0.616 | 0.7151 |
| HIV | 14 (0.42) | 3 (0.46) | 3 (0.39) | 8 (0.41) | 0.8466 | 0.8723 | 0.9423 |
| Chronic renal failure | 530 (15.83) | 101 (15.51) | 119 (15.62) | 310 (16.02) | 0.9579 | 0.76 | 0.7963 |
| Nephrotic syndrome | 40 (1.19) | 9 (1.38) | 10 (1.31) | 21 (1.09) | 0.9091 | 0.5401 | 0.6184 |
| Leukemia | 122 (3.64) | 25 (3.84) | 22 (2.89) | 75 (3.88) | 0.3193 | 0.9674 | 0.2144 |
| Hodgkin disease | 6 (0.18) | 0 (0.00) | 1 (0.13) | 5 (0.26) | 0.3552 | 0.1942 | 0.528 |
| Lymphoma | 121 (3.61) | 20 (3.07) | 30 (3.94) | 71 (3.67) | 0.3805 | 0.4745 | 0.7416 |
| Generalized malignancy | 2074 (61.95) | 413 (63.44) | 464 (60.89) | 1197 (61.86) | 0.3251 | 0.4718 | 0.6417 |
| Iatrogenic immunosuppression | 630 (18.82) | 114 (17.51) | 149 (19.55) | 367 (18.97) | 0.3255 | 0.4092 | 0.727 |
| Solid organ transplant | 411 (12.28) | 80 (12.29) | 100 (13.12) | 231 (11.94) | 0.6391 | 0.8119 | 0.3983 |
| Multiple myeloma | 80 (2.39) | 15 (2.30) | 21 (2.76) | 44 (2.27) | 0.5911 | 0.9643 | 0.4624 |
| Other | 15 (0.45) | 3 (0.46) | 1 (0.13) | 11 (0.57) | 0.2451 | 0.7461 | 0.1245 |
| **Median Charlson Comorbidity Index** | 5 | 5 | 4 | 5 |  |  |  |
| Mean Charlson Comorbidity Index (SD) | 5.44 (3.38) | 5.46 (3.40) | 5.05 (3.29) | 5.58 (3.40) | **0.0203** | 0.4302 | **0.0002** |
| *Comparisons between study clinics (e.g. A vs B) were made with Chi-square tests or ANOVA, as appropriate. | | | | | | | |
