# Supplementary material for: Impact of a guideline-based best practice alert on pneumococcal vaccination rates in adults in a primary care setting
Source: BMC Health Serv Res. 2019 Jul 10;19:474. doi: 10.1186/s12913-019-4263-2 (PMC6621991; doi:10.1186/s12913-019-4263-2)
Supplement: Supplementary file 2 — Figure S1. Vaccination Rates for At-Risk Adults Age 19–64 Years by Clinic Group and Overall. Description: The vaccination rates of at-risk adults age 19–64 years by clinic group and overall over the three time periods studied. (DOCX 59 kb) [file 12913_2019_4263_MOESM2_ESM.docx]

Additional file 2

**Figure S1. Vaccination Rates for At-Risk Adults Age 19-64 Years by Clinic Group and Overall**

***

***

FM-A = Family Medicine Group A; FM-B = Family Medicine Group B; IM-C = Internal Medicine Group C.

P<0.001 for each clinic group comparison across time

*P<0.01 vs Family Medicine Clinics Group A and B
