# Supplementary material for: Impact of a guideline-based best practice alert on pneumococcal vaccination rates in adults in a primary care setting
Source: BMC Health Serv Res. 2019 Jul 10;19:474. doi: 10.1186/s12913-019-4263-2 (PMC6621991; doi:10.1186/s12913-019-4263-2)
Supplement: Supplementary file 4 — Figure S3. Vaccination Rates for High-Risk Adults Age 19–64 Years by Clinic Group and Overall. Description: The vaccination rates of high-risk adults age 19–64 years by clinic group and overall over the three time periods studied. (DOCX 60 kb) [file 12913_2019_4263_MOESM4_ESM.docx]

Additional file 4

**Figure S3. Vaccination Rates for High-Risk Adults Age 19-64 Years by Clinic Group and Overall**

***

** ^*

***

FM-A = Family Medicine Group A; FM-B = Family Medicine Group B; IM-C = Internal Medicine Group C.

P<0.001 for each clinic group comparison across time

*P<0.01 Family Medicine Group A vs. Internal Medicine Group C; ^ P<0.05 Family Medicine Group B vs. Internal Medicine Group C
