# Supplementary material for: Impact of a guideline-based best practice alert on pneumococcal vaccination rates in adults in a primary care setting
Source: BMC Health Serv Res. 2019 Jul 10;19:474. doi: 10.1186/s12913-019-4263-2 (PMC6621991; doi:10.1186/s12913-019-4263-2)
Supplement: Supplementary file 5 — Figure S4. Intervention Effect on Pneumococcal Vaccination Rates: Implementing BPA and/or Workflow Redesign versus Health Maintenance Notifications – High-Risk Patients age 19–64 Years. Description: Pneumococcal vaccination rates in high-risk patients age 19–64 years in clinics implementing BPA and/or workflow redesigns, versus health maintenance notifications. (DOCX 30 kb) [file 12913_2019_4263_MOESM5_ESM.docx]

Additional file 5

**Figure S4: Intervention Effect on Pneumococcal Vaccination Rates: Implementing BPA and/ or Workflow Redesign versus Health Maintenance Notifications – High-Risk Patients age 19-64 Years**

BPA = best practice alert; HM = health maintenance notifications; WF = Workflow redesign.
