# Supplementary material for: Impact of a guideline-based best practice alert on pneumococcal vaccination rates in adults in a primary care setting
Source: BMC Health Serv Res. 2019 Jul 10;19:474. doi: 10.1186/s12913-019-4263-2 (PMC6621991; doi:10.1186/s12913-019-4263-2)
Supplement: Supplementary file 6 — Figure S5. Vaccination Rates for Immunocompetent Adults Aged 65+ by Clinic Group and Overall. Description: The vaccination rates of immunocompetent adults age 65+ years by clinic group and overall over the three time periods studied. (DOCX 54 kb) [file 12913_2019_4263_MOESM6_ESM.docx]

Additional file 6

**Figure S5. Vaccination Rates for Immunocompetent Adults Aged 65+ by Clinic Group and Overall**

FM-A = Family Medicine Group A; FM-B = Family Medicine Group B; IM-C = Internal Medicine Group C.

P<0.01 for pair-wise clinic comparisons for all time periods

P≤ 0.001 for each clinic group comparison across time
