# Supplementary material for: Impact of a guideline-based best practice alert on pneumococcal vaccination rates in adults in a primary care setting
Source: BMC Health Serv Res. 2019 Jul 10;19:474. doi: 10.1186/s12913-019-4263-2 (PMC6621991; doi:10.1186/s12913-019-4263-2)
Supplement: Supplementary file 7 — Figure S6. Intervention Effect on Pneumococcal Vaccination Rates: Implementing Workflow Redesign and/or BPA Versus Comparison Clinics based on Difference in Difference Analyses - Immunocompetent Patients aged 65+. Description: Pneumococcal vaccination rates in immunocompetent patients age 65+ years in clinics implementing workflow redesign and/or BPA, versus comparison clinics based on difference in difference analyses. (DOCX 31 kb) [file 12913_2019_4263_MOESM7_ESM.docx]

Additional file 7

**Figure S6: Intervention Effect on Pneumococcal Vaccination Rates: Implementing Workflow Redesign and/or BPA Versus Comparison Clinics based on Difference in Difference Analyses - Immunocompetent Patients aged 65+**

BPA = best practice alert; HM = health maintenance notifications; WF = Workflow redesign.
