# Supplementary material for: Impact of a guideline-based best practice alert on pneumococcal vaccination rates in adults in a primary care setting
Source: BMC Health Serv Res. 2019 Jul 10;19:474. doi: 10.1186/s12913-019-4263-2 (PMC6621991; doi:10.1186/s12913-019-4263-2)
Supplement: Supplementary file 8 — Figure S7. Vaccination Rates for Immunocompromised Adults Aged 65+ by Clinic Group and Overall. Description: The vaccination rates of immunocompromised adults age 65+ years by clinic group and overall over the three time periods studied. (DOCX 64 kb) [file 12913_2019_4263_MOESM8_ESM.docx]

Additional file 8

**Figure S7. Vaccination Rates for Immunocompromised Adults Aged 65+ by Clinic Group and Overall**

*^#,^ ***

***

*^^,^ ***

***

FM-A = Family Medicine Clinics Group A; FM-B = Family Medicine Clinics Group B; IM-C = Internal Medicine Clinics Group C.

P≤ 0.001 for each clinic group comparisons across time periods.

*pair-wise comparisons in time period - P<0.05 Family Medicine Clinics Group A vs. B, **Pp<0.01 vs. Family Medicine Clinics Group B, ^P<0.01 vs. Family Medicine Clinics Group A, ^#^P<0.05 vs Family Medicine Clinics Group A.
