# Supplementary material for: Impact of a guideline-based best practice alert on pneumococcal vaccination rates in adults in a primary care setting
Source: BMC Health Serv Res. 2019 Jul 10;19:474. doi: 10.1186/s12913-019-4263-2 (PMC6621991; doi:10.1186/s12913-019-4263-2)
Supplement: Supplementary file 10 — Figure S9. Pneumococcal Vaccination Series Completion by Intervention Category. Description: Pneumococcal vaccination series completion by intervention category, including no intervention, and all three interventions overall. (DOCX 59 kb) [file 12913_2019_4263_MOESM10_ESM.docx]

Additional file 10

**Figure S9. Pneumococcal Vaccination Series Completion by Intervention Category**

HM = Health Maintenance; BPA = Best Practice Alert.
